# *VvSUN* may act in the auxin pathway to regulate fruit shape in grape

**DOI:** 10.1093/hr/uhac200

**Published:** 2022-09-06

**Authors:** Huan Zheng, Yang Dong, Huilan Nong, Liyuan Huang, Jing Liu, Xin Yu, Yaguan Zhang, Lina Yang, Ben Hong, Wu Wang, Jianmin Tao

**Affiliations:** College of Horticulture, Nanjing Agricultural University, Nanjing 210095, China; College of Horticulture, Nanjing Agricultural University, Nanjing 210095, China; College of Horticulture, Nanjing Agricultural University, Nanjing 210095, China; College of Horticulture, Nanjing Agricultural University, Nanjing 210095, China; College of Horticulture, Nanjing Agricultural University, Nanjing 210095, China; College of Horticulture, Nanjing Agricultural University, Nanjing 210095, China; College of Horticulture, Nanjing Agricultural University, Nanjing 210095, China; Charles River Laboratories International, Inc., Michigan, 49071, USA; College of Horticulture, Nanjing Agricultural University, Nanjing 210095, China; Institute of Botany, Jiangsu Province and Chinese Academy of Sciences, Nanjing 210014, China; College of Horticulture, Nanjing Agricultural University, Nanjing 210095, China

## Abstract

Fruit shape is an essential agronomic feature in many crops. We identified and functionally characterized an auxin pathway-related gene, *VvSUN*. *VvSUN*, which belongs to the *SUN/IQ67-DOMAIN* (*IQD*) family, localizes to the plasma membrane and chloroplast and may be involved in controlling fruit shape through auxin. It is highly expressed in the ovary, and the expression level 1 week before the anthesis stage is positively correlated with the fruit shape index. Functional analyses illustrated that *VvSUN* gene overexpression in tomato and tobacco plants changed fruit/pod shape. The *VvSUN* promoter directly bound to *VvARF6* in yeast and activated ß-glucuronidase (GUS) activity by indole-3-acetic acid (IAA) treatments in grapevine leaves, indicating that VvSUN functions are in coordination with auxin. Further analysis of *35S::VvSUN* transgenic tomato ovaries showed that the fruit shape changes caused by *VvSUN* were predominantly caused by variations in cell number in longitudinal directions by regulating endogenous auxin levels via polar transport and/or auxin signal transduction process variations. Moreover, enrichment of the *35S::VvSUN* transgenic tomato differentially expressed genes was found in a variety of biological processes, including primary metabolic process, transmembrane transport, calcium ion binding, cytoskeletal protein binding, tubulin binding, and microtubule-based movement. Using weighted gene co-expression network analysis (WGCNA), we confirmed that this plant hormone signal transduction may play a crucial role in controlling fruit shape. As a consequence, it is possible that *VvSUN* acts as a hub gene, altering cellular auxin levels and the plant hormone signal transduction pathway, which plays a role in cell division patterns, leading to anisotropic growth of the ovary and, ultimately, an elongated fruit shape.

## Introduction

Fruits are the most valuable produce of horticultural crops. The size and shape of the fruit are crucial selection features in the course of developing new cultivars in the breeding process [1]. Wild fruits are usually small and round. Cultivars with varying fruit shapes and sizes have emerged as a result of gradual selective breeding and domestication [2]. Inheritance studies reveal that these traits are quite complex and are determined by multiple loci [3]. Researchers have undertaken comprehensive investigations on fruit shape and size as a vital criterion in the breeding of new cultivars to fulfill particular market demands, and a number of advancements have been made as a result of their efforts [4]. In recent decades, we have witnessed the cloning of several major quantitative trait loci (QTLs) related to fruit/grain size or shape in tomato [4, 5], papaya [5], cucumber [6], melon [7], peach [8], watermelon [9], cucurbits [10], rice [11, 12], and so on.

QTLs identified in tomato are perhaps the best characterized for any fruit species; the *ovate* and *sun* loci influence elongated shapes, whereas the *locule number* (*lc*) and *fasciated* (*fas*) loci both modify locule number, and both influence the shape [13]. Of these QTLs, *sun* was identified as the primary locus influencing the elongated shape of the tomato fruit, explaining up to 58% of the phenotypic variation. As speculated by Xiao *et al*. [14], the origin of the locus was a consequence of a unique 24.7-kb gene duplication activity facilitated by the long terminal repeat retrotransposon rider. Fine mapping and cloning indicate the *SUN* gene as an affiliate of the IQ67 domain-containing family [14]. The plant-specific *SUN/IQ67-DOMAIN* (*IQD*) family has been identified as modulating the shape of fruits/grains among a variety of plant species. Fine mapping of a large *F*_2_ population of cucumber led to the identification of a putative gene, *CsSUN*, which is a homologous *SUN* gene for tomato fruit shape. Gene expression analysis has indicated that the long fruit expresses much more *CsSUN* as opposed to the round fruit [15, 16]. *CmSUN-14*, a homologous gene of *CsSUN*, could play a role in the development of melon fruit shape [16]. In watermelon, a 159-bp deletion mutation in the *ClFS1* gene, which encodes the IQD protein, is crucial for determining the shape of the fruits [9]. *OsIQD14* has been identified as a critical component in modulating microtubule reconfigurations in rice hull cells and, consequently, grain shape [12]. These findings suggested that the *SUN*/*IQD-*induced fruit shape may be modulated via a conserved mechanism [12]. Overexpression of *SUN* in tomato resulted in highly elongated parthenocarpic fruits as well as twisted leaf and stem axes. Additionally, the extent of elongation is positively linked to the level of *SUN* gene expression [4, 14]. Despite the fact that *SUN* has no substantial impact on fruit weight, it does influence tomato fruit morphology by enhancing longitudinal cell division and attenuating transverse fruit cell division [4].

Auxin performs an instrumental modulatory function in the regulation of cell division and expansion and cell identity establishment [17]. Microtubule dynamics were suggested to be influenced by auxin and to have roles in controlling post-embryonic division orientation [18] or cell shape [12]. Recent studies showed that IQD proteins may be involved in controlling cell shape or cell division by modulating auxin-mediated microtubule behavior [18]. Although plant phenotypes associated with elevated *SUN* expression levels indicated a role for auxin in controlling fruit shape [13], auxin level did not change dramatically in *SUN* as opposed to wild-type fruit. Further study found that *SUN* is linked to Ca^2+^ signaling and alters auxin signal transduction gene expression, demonstrating that *SUN* might influence fruit/ovary shape by modulating the auxin-associated gene expression level in the early phase of ovary formation [19].

Grape (*Vitis* L.) is amongst the most frequently produced fruit crops and it is characterized by a broad range of fruit sizes and shapes. Wild germplasm and wine grapes are usually circular or nearly circular. Modern domesticated table grapes have much more diverse shapes, including heart-shaped, ovoid, circular, narrow ellipsoid, nearly circular, obovoid, broad ellipsoid, and cylindrical [20]. The improvement of people’s living conditions has resulted in a greater emphasis on grape quality, both in the flavor and the aesthetic aspects. The cultivation and sale of fruit varieties with unusual fruit shapes have the potential to significantly increase economic advantages. Thus, the promotion of berry quality features, as well as the discovery of the genetic pathways that influence them, have gained considerable attention. To date, numerous QTLs and potential genes associated with berry weight and size have been genetically studied in grapevine [20]. However, research on berry shape mainly focuses on physiological aspects [1]; the genetic mechanism of this diversity remains unknown, although berries have been reported to exhibit a wide range of phenotypic diversity in shape.

In this investigation, we discovered that VvSUN is a protein that has an IQ domain, localizes to the plasma membrane and chloroplast, and is highly expressed in the ovary 1 week before the anthesis stage. More importantly, the expression level is positively correlated with the fruit shape index (FSI). *VvSUN* overexpression in tomato and tobacco led to an elongated fruit/pod shape. We further showed that *VvARF6* interacts with the promoter of *VvSUN* in yeast and that *VvSUN* activity was triggered by indole-3-acetic acid (IAA) treatment *in vitro*. Moreover, the IAA level and auxin-related genes were significantly altered in *35S::VvSUN* transgenic tomato lines. Combined weighted gene co-expression network analysis (WGCNA) and Kyoto Encyclopedia of Genes and Genomes (KEGG) analysis confirmed that the plant hormone signal transduction pathway may have an important role in controlling fruit shape. Our results offer a novel perspective on the function performed by *VvSUN* in modulating the elongated fruit shape in the auxin pathway during the early phases of fruit growth.

## Results

### Phylogenetic and transcriptional profiling analysis of *VvSUN*s

**Figure 1:**
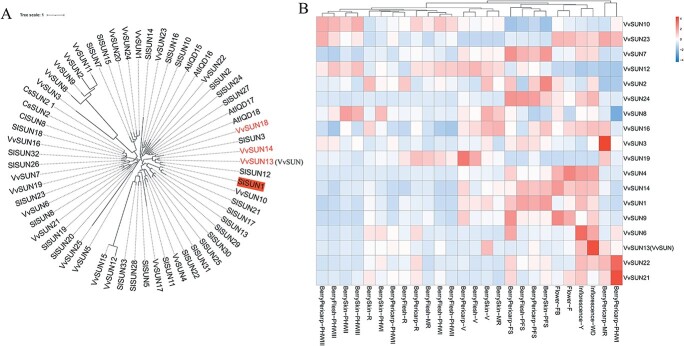
Phylogenetic and transcriptional profiling analysis of *VvSUN*s*.* (A) Investigation of the phylogenetic link of VvSUN proteins from grapevine with *A. thaliana*, *Cucumis sativus*, *Citrullus lanatus*, *C. lanatus*, and *Solanum lycopersicum*. A phylogenetic tree was created utilizing the neighbor-joining method in MEGA11 incorporating 61 SUN amino acid sequences discovered in various species, including predicted full-length VvSUNs from the grapevine. (B) Expression profiles of the *VvSUN* genes in grape tissues. Transcriptomic data were collected from the NCBI (SRA database, NO:GSE36128) [23]. The gene expression values were transformed using log_10_ base to provide a heat map representation using the Tutools platform (http://www.cloudtutu.com/). Y, young; MR, mid-ripening; WD, well developed; F, flowering; FB, flowering begins; PFS, post-fruit-set; V, veraison; PHWI, pericarp post-harvest withering I (1st month); PHWII, post-harvest withering II (2nd month); PHWIII, post-harvest withering III (3rd month).

A BLASTP search of the *Vitis vinifera* genome using 33 *Solanum lycopersicum* SUN protein sequences as a query identified a total of 25 putative *SUN* genes (*VvSUN*s). Twenty-five genes were named *VvSUN1*–*VvSUN25* according to their physical locations on the chromosomes. The phylogenetic tree was constructed with predicted *S. lycopersicum* SUNs, VvSUNs and other species’ SUN protein sequences using a neighbor-joining algorithm in MEGA11 with 1000 bootstrap replicates (Fig. 1A). VvSUN13 (LOC100253695), VvSUN14 (LOC100265924), and VvSUN18 (LOC100256816) were classified into the same subgroup as SlSUN1, indicating that these genes might share similar functions. SlSUN1 was identified as SUN, and its role in fruit shape control has been widely studied [14, 21]. Further study through PlantDGD (http://pdgd.njau.edu.cn:8080) online software revealed that *VvSUN13* and *VvSUN14* are a duplicate gene pair [22], both of them located on chromosome 8.

Expression profiling of *VvSUN*s related to flowers and berries were analyzed by using the previously published grape (*V. vinifera* cv. ‘Corvinathe’) RNA-sequencing data from NCBI (accession number GSE36128) [23]. *VvSUN13* showed high expression in young and well-developed inflorescences, the pericarp at mid-ripening stage, and berry skin at veraison stage, while *VvSUN14* was expressed constantly from young inflorescences to flowering flowers and also in berries (skin, pericarp, and flesh) at post-fruit-set stage (Fig. 1B). These duplicate gene pairs that have diverged in their expression level indicated that their function might alter during evolution. *VvSUN18* is not present in Fig. 1B because of the lack of corresponding expression data. *VvSUN13*, *VvSUN14*, and *VvSUN18* were considered as the candidate genes in controlling fruit shape for further research.

### Morphological assay and expression analysis of *VvSUN*

**Figure 2:**
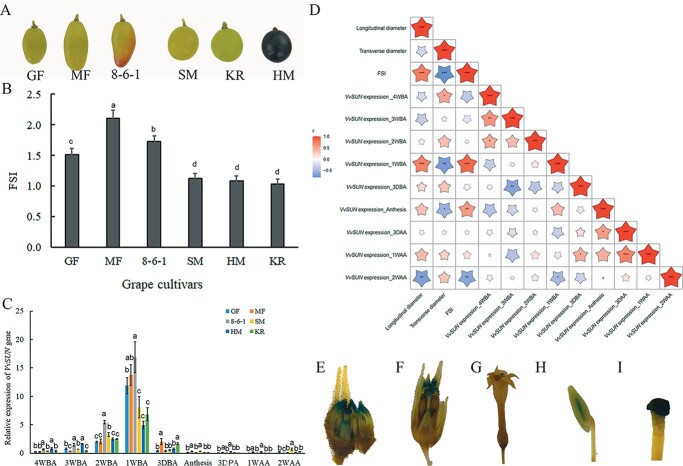
Expression pattern of the *VvSUN* gene. (A) Representative grape varieties with different shapes. MF, ‘Minicure Finger’; GF, ‘GoldFinger’; KR, ‘Kourgan Rose’, 8-6-1 (‘Beni Pizzutello’ seedling); HM, ‘Houman’; SM, ‘Shine-Muscat’. (B) Fruit shape index (FSI; length/diameter ratio) analysis of mature berries of different grape varieties. Lower-case letters (*P* ≤ .01) represent a significant difference among different types, as determined by Student’s *t*-test. (C) Transcription of *VvSUN* at various phases of ovary/fruit development was identified utilizing qRT–PCR. Normalization of the expression levels was conducted using the *VvActin* transcript, and *VvSUN* expression in HM at the 4 WBA stage was set as the control group to compute relative expression levels. Experimental studies were replicated biologically three times, and the data are presented as the mean ± standard deviation. Lower case letters (*P* ≤ .01) represent a significant difference among different stages, as determined by Student’s *t*-test. (D) Correlation coefficient matrix between mature grape morphological parameters and *VvSUN* expression levels at different ovary/fruit stages. Star size represents the Pearson correlation index. ^***^P < .001; ^**^P < .01; ^*^P < .05. (E–I) Analysis of promoter–GUS fusion illustrating *VvSUN* expression in ovaries, stigmas, and young petals before tobacco anthesis. However, only anthers and stigmas were observed to be stained by GUS at the anthesis stage.

Xiao *et al*. [14] discovered that the *SUN* transcription factor plays a role in the modulation of tomato fruit shape. Mature fruits harvested from six different grape cultivars were utilized to study the potential relationship between the level of *VvSUN* gene expression and fruit morphology (Fig. 2A), measure their longitudinal diameter and length (Supplementary Data Fig. S1A and B), and derive the FSI (length/diameter ratio) (Fig. 2B). The FSIs of ‘Gold Finger’ (GF),‘Minicure Finger’ (MF), and 8-6-1 (‘Beni Pizzutello’ seedling) were much higher than those of ‘Shine-Muscat’ (SM), ‘Kourgan Rose’ (KR), and ‘Houman’ (HM) (Fig. 2B).

Different cultivars were used to assess the levels of *VvSUN13*, *VvSUN14*, and *VvSUN18* mRNA transcripts in their ovary and young fruit growth phases at various periods (Fig. 2C, Supplementary Data Fig. S1C and D). *VvSUN13* showed consistency in expression patterns among all the cultivars. It gradually increased in all cultivars 2 weeks before the anthesis (WBA) stage and reached the highest expression level at the 1 WBA stage; after that, the transcript levels were sharply decreased 3 days before the anthesis (DBA) stage and remained low until 2 weeks after the anthesis (WAA) stage. Moreover, the expression of *VvSUN* in GF, MF, and 8-6-1 at 1 WBA was much higher than in other cultivars. However, both *VvSUN14* and *VvSUN18* showed no direct relationship between the levels of *VvSUN* gene expression and fruit morphology (Supplementary Data Fig. S1C and D). Moreover, sequence comparisons revealed that VvSUN13 was most closely related to SlSUN, with 43.57% identity for the complete protein sequence. Therefore, we predicted the *VvSUN13* gene as the corresponding grapevine *SUN* ortholog, which may also be involved in controlling fruit shape. Since *VvSUN13* was first studied as *VvSUN* by Zhang *et al*. [24], we also refer to *VvSUN13* as *VvSUN* in the present article.

In accordance with these parameters measured above, the correlation coefficients between fruit shape parameters and the relative expression of *VvSUN* were calculated. The *VvSUN* expression level at the 1 WBA stage was positively correlated with the longitudinal length of the fruit and showed the highest correlation to FSI (0.87) (Fig. 2D).

We additionally analyzed the expression profiles by fusing the *VvSUN* promoter region to the ß-glucuronidase (GUS) reporter (Supplementary Data Fig. S2). GUS staining was visible in the ovaries, anthers, stigmas, and young petals (Fig. 2E–I). These findings indicate that although *VvSUN* is predominantly expressed in the young ovaries, it may also function in other tissues, mostly those in which many cell divisions occur.

A conserved domain search confirmed that VvSUN protein has the IQ67 domain, a conserved core section of 67 amino acids that are involved in the recruitment of calmodulin or function as a Ca^2+^ sensor. There are two distinct categories of IQ67 domains: one is the Ca^2+^-independent IQ motif, the IQ motif (I/L/VQxxxRxxxxR/K or IQxxxRGxxxR); the other one is the Ca^2+^-dependent IQ motifs, the 1-8-14 motifs (1-4 [FILVW]x_6_[FAILVW]x_5_[FILVW]) and 1-5-10 (1–4 [FILVW]x_3_[FILV]x_4_[FILVW]) [25]. The IQ67 domain of VvSUN contains two IQ motifs (amino acid residues 109–123 and 135–145), three 1-5-10 motifs, and two 1-8-14 motifs (Supplementary Data Fig. S3).

Our results revealed that the elongated fruit shape was positively correlated with the high expression level of *VvSUN* at the 1 WBA stage, but what induced the *VvSUN* expression level variation is still unknown. To subsequently examine the genetic mechanisms for these differences in expression, we separately cloned and sequenced the cDNA and promoter sequences (from −1833 bp to ATG) of the *VvSUN* gene from these six grape cultivars. Sequence alignment analysis showed no direct link between the SNPs and fruit shape (Supplementary Data Figs S4 and S5), indicating that the *VvSUN* coding sequences and −1833 bp upstream cannot explain expression variation.

### Overexpression of *VvSUN* leads to altered plant architecture

**Figure 3:**
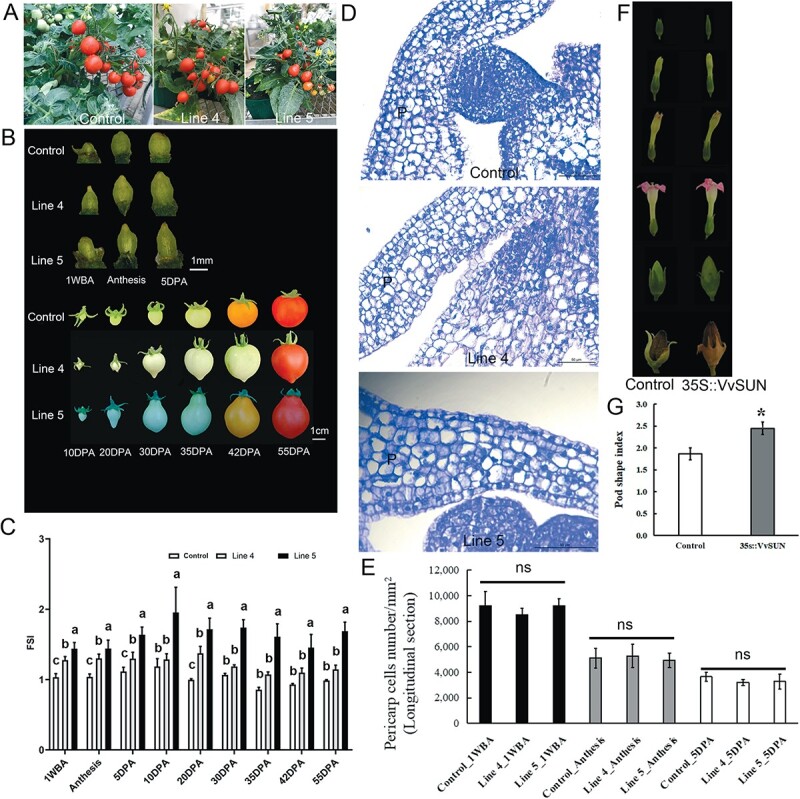
Phenotypic characteristics of *35S::VvSUN* plants. (A) Tomato plants of control and *35S::VvSUN* transgenic lines 4 and 5. (B) Main stages of tomato fruit development. (C) Changes in FSI during fruit development. Lower-case letters represent a significant difference (*P* ≤ .01) among different genotypes, as demonstrated by Student’s *t*-test. (D) Microscopic analysis of a longitudinal section of the pericarp at the 1 WBA stage. Scale bars = 50 μm. The pericarp (p) is indicated. (E) Comparison of pericarp cell numbers in control and lines 4 and 5 at 1 WBA, anthesis, and 5 DPA stages. (F) Tobacco flowers and pods of control and *35S::VvSUN* transgenic plants. (G) Comparison of pod shape index in control and *35S::VvSUN* transgenic tobaccos. The mean standard deviation is indicated by the error bar. Student’s *t*-test was used to determine statistical significance. ^*^P ≤ .05; ns, not significant.

To verify the role of the *VvSUN* gene in the control of fruit shape, transgenic tomato lines were generated in which the *VvSUN* gene was overexpressed under the control of the cauliflower mosaic virus (CaMV) 35S promoter. From the five independent *T*_4_ generations of transgenic tomato lines that were produced, two *VvSUN* overexpression lines (lines 4 and 5) with the highest expression were selected for subsequent investigation (Supplementary Data Fig. S6A, Fig. 3A). The ovaries and fruits were obtained from the control and two overexpressing lines, and their longitudinal length, diameter, and shape index were determined (Fig. 3B and C, Supplementary Data Fig. S6B and C). The differences in length, width, and shape index were firstly observed in 1 WBA ovaries, and it was found that each genotype’s FSI did not alter much with the variation in days post-anthesis (DPA) and that it remained constant from 1 WBA till the mature stage (Fig. 3C). This shows that the shape of the fruit has already been decided during the early stages of ovary development.

On the sliced paraffin section three distinct regions were identified: the pericarp, the columella, and the placenta (Supplementary Data Fig. S7). Cells in these regions steadily increased in size from 1 WBA to 5 DPA. Nonetheless, at any stage of development, the cell size and shape of transgenic tomatoes were comparable to those of the control (Fig. 3D). We further checked the cell numbers (number/mm^2^) in the pericarp both in longitudinal section and cross-section (Fig. 3E, Supplementary Data Fig. S8A), as well as cell shapes at 1 WBA stage, and the results showed no significant difference between transgenic tomatoes and control (Supplementary Data Fig. S8B–G), which implies that the elongated fruit morphology of lines 4 and 5 was mostly attributable to the creation of more cells in the longitudinal axis as a result of the increased rate of cell division.

To further validate the function of *VvSUN* for elongated fruits, we also introduced *35S::VvSUN* into tobacco (K326). Twelve putative transgenic lines were chosen on a medium that contained 30 mg l^−1^ hygromycin and confirmed by qRT–PCR (Supplementary Data Fig. S9A). Significant differences in pod morphology were discovered in tobacco plants that constitutively expressed *VvSUN*, with transgenic pods exhibiting a longer pod length, shorter pod width, and a higher pod shape index compared with pods from control plants (Fig. 3F and G, Supplementary Data Fig. S9). Interestingly, we also noticed that the transgenic tobacco plants had longer leaf rachises and an increased leaf shape index compared with the control plants. Cell forms and sizes in the lower epidermis leaves of transgenic tobacco were very similar to that in the wild type (Supplementary Data Fig. S5). Therefore, these findings also indicate that longer leaves contain more cells.

### The *VvSUN* promoter interacts with *VvARF6* in yeast and exhibits stronger auxin-induced activity

**Figure 4:**
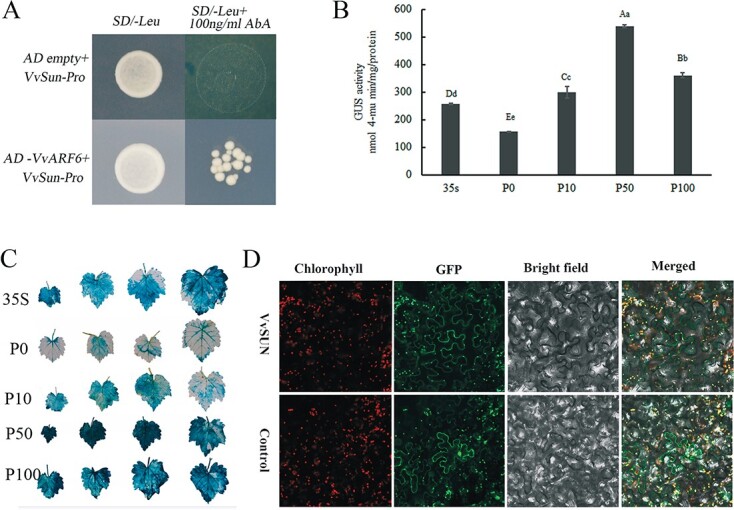
Analysis of the *VvSUN* promoter and location analysis of *VvSUN*. (A) *VvARF6* interfaces with the *VvSUN* promoter, as shown by a Y1H assay. As bait, Y1H strains containing the promoters of VvSUN were employed in the experiment. pGADT7 was set as the vector for the control. (B) Measurement of GUS expression levels in grapevine leaves was accomplished based on the transient expression of the VvSUN promoter in response to varying dosages of IAA treatment. The *35S* promoter acted as the positive control. Outcomes are presented as the means (± standard deviation) of three biological experiments. Lower-case letters (*P* ≤ .01) and capital letters (*P* ≤ .05) represent a significant difference among different treatments, as determined by Student’s *t*-test. (C) Histochemical staining of GUS function mediated by the *VvSUN* promoter under different concentrations of IAA treatment in grapevine leaves. The 35S promoter is the control and P0, P10, P50, and P100 represent the 0, 10, 50, and 100 mg/l IAA treatments, respectively. (D) Subcellular distribution of *VvSUN* in the leaves of *N. benthamiana* that had been transiently converted. 35S-GFP was adopted as the control, and expression was monitored utilizing a confocal laser scanning microscope. Chlorophyll channel (left 1), GFP channel (left 2), bright field (right 2), and merged images (right 1) are shown.

Numerous *IQD* genes in *Arabidopsis* are potential targets of *ARF5*, an early auxin-responsive factor, and the *AtIQD15* expression level is elevated following exogenous auxin application [17]. Interestingly, *in silico* analysis of the *cis* elements present in the −1833-bp promoter sequence of the *VvSUN* genes revealed numerous motifs (Supplementary Data Table S1), including an ARFAT element that functions as an ARF binding site. Our previous research found that *VvARF6* (LOC100242923) was activated by exogenous plant hormone treatment (not published data). To determine whether VvARF6 interacts with the *VvSUN* promoter, we examined the interactions between VvARF6 and the *VvSUN* promoter using a yeast one-hybrid (Y1H) assay. The Y1H results demonstrated that the VvARF6 protein interacted with the *VvSUN* promoter fragment, confirming that the VvARF6 protein recognizes the *cis* element in the *VvSUN* promoter in yeast (Fig. 4A).

For the purpose of determining whether the *VvSUN* gene could be triggered by auxin, we transiently transformed the *VvSUN* promoter into grape leaves and measured the GUS activity in leaves treated with 0, 10, 50, and 100 mg/l of auxin. Compared with the mock control (*35S::GUS*), GUS activity mediated by the *VvSUN* promoter was significantly increased when exogenous auxin was applied, and the highest GUS activity was achieved at 50 mg/l IAA treatment (Fig. 4B and C).

### Subcellular localization of VvSUN

We also transiently generated the *GFP-VvSUN* fusion protein controlled by the 35S promoter in *Nicotiana benthamiana* leaves and recorded its cell localization utilizing confocal laser scanning microscopy to establish the subcellular location where *VvSUN* operates. By overlapping the fluorescence of GFP and chlorophyll, strong fluorescence of the GFP-VvSUN fusion protein was identified in the plasma membrane and chloroplast (Fig. 4D).

### The *VvSUN* gene affects auxin pathways

**Figure 5:**
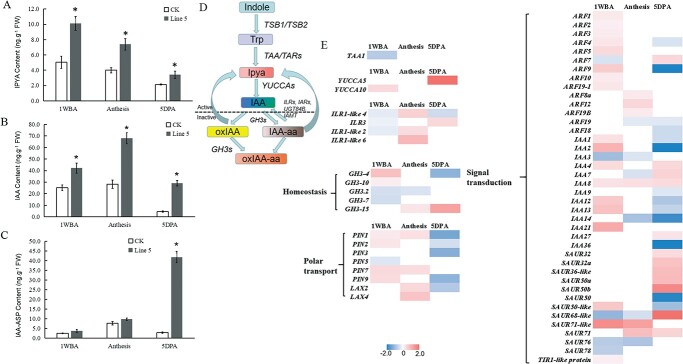
The *VvSUN* gene affects auxin pathways. Comparative analysis of (A) IAA precursors (IPYA), (B) free auxin (IAA), and (C) IAA conjugates (IAA-ASP) changes between the *35S::VvSUN* transgenic tomato and control at different fruit development stages. Each value represents the mean ± standard deviation of three repetitions. FW, fresh weight. (D) Simplified diagram of IAA metabolism. (E) Heat map for each class indicates the expression changes of different auxin-related genes. The color legend represents the log_2_-fold change of the VvSUN/control ratio. A positive value means that the gene is significantly up-regulated in the transgenic plants and a negative value means that the gene is significantly down-regulated in the transgenic plants.

Previous studies on IQD family proteins hypothesized possible links to auxin pathways [14], and the fruit shape phenotype of *SUN*-overexpressing plants is comparable to the shape of auxin mutants [4, 26]. In this study, auxin response factor VvARF6 interacted with the *VvSUN* promoter and induced GUS activity under different IAA treatments. To determine whether the *VvSUN* gene can regulate fruit shape by modulating auxin, UHPLC–MS/MS analysis was carried out to detect and quantify auxin and auxin-related compounds, such as IAA precursors (indole-3-acetamide, IPYA), free auxin (IAA), and IAA conjugates (indole-3-acetic acid-aspartate, IAA-Asp) in *35S::VvSUN* line 5 and control at 1 WBA, anthesis and 5 DPA stages. *VvSUN* overexpression contributed to elevation in the levels of IPYA and IAA in all stages (Fig. 5A and B). IAA-Asp was also detected, but there was no significant difference between *35S::VvSUN* line 5 and control at the 1 WBA and anthesis stages. However, the *35S::VvSUN* line had significantly increased IAA-Asp at the 5 DPA stage, where the concentration was ~14.9-fold higher than in the control tomato (Fig. 5C). Collectively, the above findings imply that inactivation processes might be essential to maintain auxin homeostatic function and to prevent excessive IAA response amplification in cases where it has been initiated.

In order to acquire a deeper comprehension of the auxin pathway, we investigated the transcriptional patterns of genes that could be involved in auxin biosynthesis, homeostasis, conjugation, and auxin influx transporter by RNA-seq at distinct stages of development in *35S::VvSUN* and the wild type, as depicted in Fig. 5D and E. *VvSUN* significantly influenced the expression of 60 auxin-related genes, such as 3 auxin-biosynthesis-associated genes (*TAA1*, *YUCCA5*, and *YUCCA10*), 4 IAA-amino acid hydrolases (*ILR*s), 5 auxin homeostasis-related genes (*GH3*s), 8 polar transport genes (*PIN*s and *LAX*s), and 40 signal transduction genes (*ARF*s, *IAA*s,*SAUR*s, and *TIR1-like* gene) (Fig. 5D and E; gene_ID is listed in Supplementary Data Table S3). Regarding the interaction between auxin and *VvSUN*, we propose that *VvSUN* regulates tomato fruit shape not only by auxin levels but also by polar transport and/or auxin signal transduction processes.

### Gene expression profiles associated with *VvSUN*

**Figure 6:**
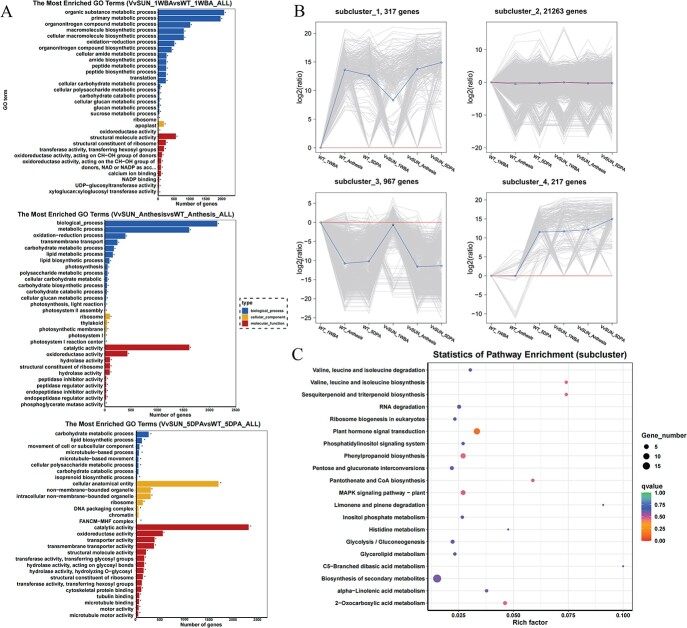
Identification of DEGs in *35S::VvSUN* transgenic tomato. (A) GO term enrichment analysis of the DEGs of ovary/fruit in *35S::VvSUN* transgenic tomato at 1 WBA, anthesis, and 5 DPA stages. ^*^P < .05. (B) Positive values were significantly higher in *35S::VvSUN* transgenic tomato. Negative values were significantly higher in the control. Cluster analysis results were visualized using a multigene line plot. The expression patterns of the DEGs were divided into groups based on the developmental time points utilizing *K*-means clustering. (C) KEGG enrichment analysis of the DEGs in *35S::VvSUN* transgenic tomato, showing contrasting expression trends in comparison with the control tomato.

To evaluate the mechanisms through which *VvSUN* modulates fruit shape, we determined the differentially expressed genes (DEGs) in pairwise comparisons of *35S::VvSUN* transgenic tomato ovary/fruits and control tomato at different stages (1 WBA, anthesis, and 5 DPA stages). Analysis between VvSUN_1WBA versus control_1WBA, VvSUN_Anthesis versus control_Anthesis, and VvSUN_5DPA versus control_5DPA showed that 2971, 2118, and 2785 genes were upregulated, respectively, and that 3128, 1927, and 2919 genes were downregulated, respectively, at three different stages (Supplementary Data Figs S10 and S11). Gene ontology (GO) term enrichment (*P* ≤ .05) analysis illustrated that these DEGs were predominantly implicated in organic substance metabolism, primary metabolic process, oxidoreductase activity, catalytic activity, metabolic process, ribosome, and so on. In addition, genes related to transmembrane transport, calcium ion binding, cytoskeletal protein binding, tubulin binding, and microtubule-based movement were also enriched (Fig. 6A). Interestingly, we found that the expression of transmembrane transport pathway (GO:0055085)-related genes was significantly changed among the three stages (Supplementary Data Fig. S12; gene_ID is listed in Supplementary Data Table S4).

We further clustered the DEGs on the basis of the log_2_-fold change in *35S::VvSUN* and control utilizing the *K*-mean cluster. There were four patterns detected in the time-series gene expression profiles, which were then displayed utilizing a multigene line plot (Fig. 6B). The same DEG subclusters in two different genotypes showed different expression patterns and were considered the main genes regulated by the *VvSUN* gene. In subclusters 1 and 4, there were 317 and 217 DEGs, respectively, that exhibited contrasting expression trends. KEGG enrichment analysis showed a remarkable enrichment of these two subclusters in the plant hormone signal transduction pathway (Fig. 6C). In the GO analysis of clusters 1 and 4 (Supplementary Data Fig. S13), pathways were most related to the DNA metabolic process, cysteine-type peptidase activity, and mRNA maturation, such as spliceosomal snRNP assembly, SMN complex, ribonucleoprotein complex assembly, and ribonucleoprotein complex subunit organization.

### WGCNA analysis identified highly connected ‘hubs’ and associated specific modules with genetic and fruit shape traits

**Figure 7:**
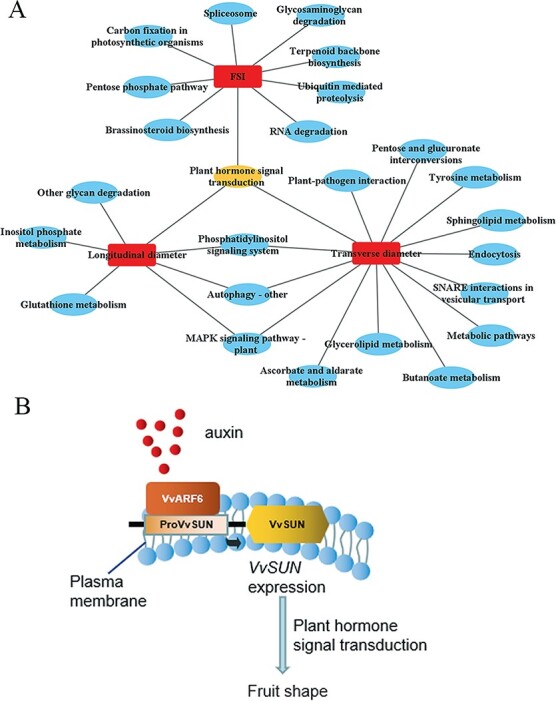
The pathway correlation network and proposed model of fruit shape control. (A) Key pathways in the control of fruit shape by WGCNA and KEGG analysis were characterized and identified, then significantly enriched pathways were used to construct a correlation network by Cytoscape. (B) Proposed model of how *VvSUN* regulates fruit shape. *VvSUN* is situated in the plasma membrane and exogenous auxin may induce expression of *VvARF6*, then *VvARF6* activates *cis*-elements in *VvSUN* promoters to induce gene expression. The increased expression of *VvSUN* stimulates endogenous auxin accumulation and polar transport and/or auxin signal transduction process variations. Therefore, we suppose that *VvSUN* may not only respond to exogenous auxin treatment but also modulate the elongated fruit shape in the plant hormone signal transduction pathway during the early phases of fruit growth.

In order to reveal gene networks associated with fruit shapes in *35S::VvSUN* transgenic tomatoes, The gene expression profiles of all these 22 510 genes were analyzed to identify gene co-expression modules using the R package WGCNA. Here, 11 co-expression modules were identified, among which the ‘green’, ‘lightcyan’, ‘darkred’, and ‘pink’ modules were not only significantly associated with fruit shape but also with auxin-related compounds (Supplementary Data Fig. S14), indicating that auxin-related genes may be correlated with the control of fruit shape. Specifically, the longitudinal diameter was positively correlated with the expression of genes in the ‘green’, ‘darkred’, and ‘pink’ modules (Supplementary Data Fig. S14), with a coefficient of 0.77 (*P* = 2e−04), 0.83 (*P* = 2e−05), and 0.6 (*P* = 0.008), respectively (Supplementary Data Fig. S14). The transverse diameter was positively correlated with the expression of genes in the ‘brown’ and ‘green’ modules with a coefficient of 0.82 (*P* = 3e−05) and 0.57 (*P* = 0.01), respectively (Supplementary Data Fig. S14). Moreover, the FSI was positively correlated with the expression of genes in the ‘grey60’, ‘lightcyan’, ‘darkred’, and ‘pink’ modules with a coefficient of 0.5 (*P* = .04), 0.5 (*P* = .03), 0.63 (*P* = .005), and 0.79 (*P* = 9e−05), respectively. Genes clustered in above modules were picked out according to the gene significance (GS) values (GS > coefficient values of the trait) and *P* values (P.GS < *P* values of the trait) for further KEGG pathway analysis. The results showed that these genes were significantly enriched in several pathways (Supplementary Data Table S5). Interestingly, the plant hormone signal transduction pathway was a jointly owned pathway by longitudinal diameter, transverse diameter, and FSI trait modules (Fig. 7A). Then, genes involved in the processes of the plant hormone signal transduction pathway in each trait module were selected to construct a gene network by Cytoscape. As seen in Supplementary Data Fig. S15, out of the 41 hormone signal transduction pathway genes, 14 were plant hormone-related genes and among them around 50% were auxin-related genes according to the annotation of genes. Therefore, it is conceivable that the mechanisms underlying plant hormone signal transduction serve as the primary hub for interactions with other pathways implicated in controlling the elongated fruit morphology that arises from *VvSUN* overexpression in the plant.

## Discussion

### *VvSUN* regulates fruit shape

The shape of the fruit is among the most distinguishing characteristics of the table grape. Nonetheless, only a few gene-oriented research reports have concentrated on the discovery of genes relevant for grape berry morphology, despite the fact that a vast spectrum of phenotypic diversity in berry shape has been reported. In this study, we cloned a grape *VvSUN* gene that encodes an IQD-like protein. The phylogenetic tree showed that VvSUN is one of the closest homologs to tomato SUN (Fig. 1A). Genes clustered in the same subclade are more likely to have similar functions [12]. Earlier research reports have demonstrated that upregulation of *SUN* contributed to the development of fruit that was very elongated and generally seedless [4]. Using anatomical findings, it was discovered that *SUN* has a significant influence on the morphology of the fruit before its anthesis, but it is after anthesis that *SUN*’s most striking influence on shape is evident, which is likely due to the changes in cell division rates in the longitudinal direction, leading to a cell number increase along the proximal–distal axis [4]. In our results, the expression levels of *VvSUN* were much higher in elongated grape cultivars than in round or near-round types at the 1 WBA stage (Fig. 2C). Moreover, the *VvSUN* expression level at the 1 WBA stage was positively correlated with the longitudinal length of the mature berry and also showed the highest correlation to FSI (0.87) (Fig. 2D). Overexpression of *VvSUN* driven by the 35S promoter in tomatoes led to an increase in the FSI in tomatoes but showed little or no impact on cell size and form (Fig. 3A–E, Supplementary Data Figs S6–S8). The function of *VvSUN* was also validated in transgenic tobacco, which showed a significant increase in the pod shape index as well as in the leaf shape index. Moreover, the cell form and sizes in lower epidermis leaves of transgenic tobacco were similar to those of the wild type (Fig. 3F and G, Supplementary Data Fig. S9). Given the difference in fruit types between tomato and tobacco, it is likely that the basic function of *VvSUN* in regulating the FSI by changing cell division is likely conserved. Furthermore, to our knowledge, VvSUN has not previously been recognized as a gene that regulates the morphology of fruits. Hence, we infer that *VvSUN* is a novel gene that modulates fruit shape by changing the cell division rate.

Our data also show that the differences in the levels and timing of *VvSUN* expression were positively correlated with the fruit shape phenotype (Fig. 2C and D). However, sequence analyses revealed that there were no consensus sequence diversities in the coding regions and −1833 bp upstream of the *VvSUN* gene between the elongated and the round grape cultivars (Supplementary Data Figs. S4 and S5), indicating that the *VvSUN* coding sequences and −1833 bp upstream cannot be the reason for expression variation. Gene regulation in multicellular eukaryotes is complex, with many layers of regulation [27], including mutations in coding sequences or promoter regions [14, 28], long-range control by distant repressors or enhancers, alteration of epigenetic states, coordinated expression of genes [29], and regulation by transcription factors, including microRNAs (miRNAs), small interfering RNAs (siRNAs), messenger RNAs (mRNAs), and non-coding RNAs [30]. In this case, it is a big challenge and needs further effort in order to decipher how variation in regulatory mechanisms eventually results in changes in *VvSUN* gene expression profiles.

### Mechanisms by which *VvSUN* controls fruit shape

The plant hormone auxin performs a fundamental function in the modulation of cell expansion, cell division, and cell identity establishment [18, 31]. Earlier research reports illustrated that IQ domain-containing proteins belong to a calmodulin-binding protein family and play a role in the regulation of fruit shape by modulating auxin signal transduction [12, 32]. In this study, we also showed that the *VvSUN* promoter interacts with grape VvARF6 in yeast (Fig. 4A), and GUS activity driven by the *VvSUN* promoter was significantly increased when exogenous auxin was applied (Fig. 4B and C). More importantly, ectopic overexpression of *VvSUN* in tomatoes not only enhanced endogenous IAA content but also remarkably influenced the expression of auxin-associated genes, especially those implicated in polar transport and signal transduction (Fig. 5E). In addition, the combination of clustering, WGCNA, and KEGG enrichment analysis demonstrated a substantial enrichment of the DEGs in *35S::VvSUN* transgenic tomatoes in the plant hormone signal transduction pathway in pairwise comparisons with control (Figs 6B and C and 7A). It has been shown that auxin signaling plays an important role in apple size [33] and the inhibition of polar auxin transport in tobacco (*Nicotiana tabacum*) changes the orientation of cell division [34, 35]. Recent studies have shown that multiple *IQD* genes are candidate *ARF5* targets and are transcriptionally regulated by auxin signaling [12, 32]. Multiple studies have demonstrated that active auxin levels and distributions are closely regulated by the actions of synthesis, inactivation, and transport. Additionally, Wang *et al.* [13] detected that *SUN* shifted the expression of auxin polar transport and signal transduction during the initial stages of the ovary’s development. Thus, it is reasonably suggested that *VvSUN* alters the longitudinal direction of cell division in fruit by affecting auxin transport or the auxin signaling pathway.

Recently, several lines of evidence have shown that auxin can influence microtubule dynamics [36], and genetically controlled microtubule depolymerization in embryos leads to the disruption of asymmetric divisions [18]. Most *IQD* members co-locate with microtubules, the cell nucleus, or membranes, which are implicated in the transduction of Ca^2+^ signals into cell responses via the modulation of a variety of target proteins [17, 37]. As a result of the elevation in cytosolic Ca^2+^ concentrations caused by auxin treatment, the activity of the IQD is regulated posttranslationally via stimulation of the Ca^2+^ CaM signaling pathway [38]. Ca^2+^ CaM, on the other hand, has an effect on auxin production by directly interfacing with components of the auxin transport and signaling mechanism, including PINOID (PID) or small auxin upmodulated RNA 19 (SAUR19) [38]. A conserved domain search confirmed that the VvSUN protein contains a Ca^2+^-dependent IQ motif (Supplementary Data Fig. S3), and we detected that *VvSUN* was localized in the plasma membrane and chloroplast (Fig. 4D). The transcriptome analysis of *VvSUN* overexpression in tomatoes revealed that DEGs were enriched in calcium ion binding, cytoskeletal protein binding, tubulin binding, and microtubule-based movement pathways (Fig. 6A). Moreover, the transmembrane transport pathway (GO:0055085) was activated in *35S::VvSUN* transgenic tomato among the three stages using pairwise comparisons with control (Supplementary Data Fig. S12). The findings presented in this work lead us to postulate that VvSUN is situated in the plasma membrane and functions as a hub gene to translocate cellular auxin and calcium signaling (Fig. 7B). This, in turn, alters the pattern of cell division, contributing to the anisotropic expansion of the ovary and the presence of a fruit with an elongated morphology.

## Materials and methods

### Plant materials and growing conditions

Grape plants (*V. vinifera* L.) of cv. ‘Minicure Finger’ (MF), ‘Kourgan Rose’ (KR), 8-6-1 (‘Beni Pizzutello’ seedling), and *V. vinifera* × *Vitis labrusca* cv. ‘GoldFinger’ (GF), ‘Houman’ (HM), and ‘Shine-Muscat’ (SM) from the Tang Shan Vineyard (College of Horticulture, Nanjing Agricultural University, Nanjing, China) were sampled during the 2020 growing season. Ovary and fruit samples were collected at 4 WBA, 3 WBA, 2 WBA, 1 WBA, 3 DBA, anthesis (when 50% of the caps were off), 3 days post anthesis (3 DPA), 1 WAA, and 2 WAA. The samples were promptly frozen in liquid nitrogen and kept at a temperature of 80°C till RNA extraction was done.

Tomato (*S. lycopersicum* Mill. cv. ‘Micro Tom’) and tobacco (*N. tabacum* L. ‘K326’) plants were cultivated in a normal greenhouse environment at Nanjing Agricultural University. The following were the conditions under which the culture chamber was set up: 16-hour day/8-hour night cycle at constant 25°C, 60% humidity, and 250 μmmol m^−2^ s^−1^ luminous intensity [39].

### Morphological analysis of grapes and transgenic plants

The fruits of grapes (the six different cultivars mentioned above) and tomato, and the tobacco pod samples were measured for fruit/pod diameter and length by using a Vernier caliper (Mitutoyo, Kawasaki, Japan) at 7, 9, 15, 20, 36, 40 DPA. The ovaries and young fruits of tomato at 1 WBA, anthesis, and 5 DPA stages were measured with a stereo microscope (SZX10, Olympus, Japan).

Histological examination of the fruits at various growth stages was accomplished by the use of paraffin segmentation. After fixing the obtained fruit samples in formaldehyde–acetic acid–ethanol (FAA) for at least 24 hours, they were washed in 50% ethanol for 10 minutes before being dried and embedded in paraffin in accordance with the conventional techniques reported by Godoy *et al*. [40].

The *VvSUN* expression level and phenotypic correlation coefficient were analyzed and visualized using online software (http://www.cloudtutu.com/).

### Identification and phylogenetic analysis of *VvSUN* genes

The IQ67 domain of SlSUN [22] was utilized to categorize members of this family in grapes. Systematic BLAST screenings were conducted on all sequence data in genome databases for grape (version 2.0) and its annotation (http://ftp.ensemblgenomes.org/pub/plants/release-46/gtf/vitis_vinifera/) based on the domain retrieved from the SOL Genomics Network (SGN, http://solgenomics.net) as initial queries. The identified protein sequences were subsequently validated for IQ67 domain components in the SMART databases (http://smart.embl-heidelberg.de/) and NCBI Conserved Domains database (https://www.ncbi.nlm.nih.gov/Structure/cdd/wrpsb.cgi). All the protein sequences containing errors or lacking the IQ67 domain were removed from the study.

A phylogenetic tree was constructed utilizing the neighbor-joining (NJ) technique in the MEGA (Molecular Evolutionary Genetics Analysis) program version 11.0, and edge support was evaluated by applying 1000 bootstrap replicates [41]. The SUN sequences of additional species, such as cucumber (*Cucumis sativus*) and watermelon, were acquired from GuGenDB (http://cucurbitgenomics.org/) and the sequences of AtIQDs were retrieved from NCBI (https://www.ncbi.nlm.nih.gov/). This study comprised sequences with the following GenBank accession codes: *V. vinifera*, VvSUN1 (LOC100267340), VvSUN2 (LOC100260828), VvSUN3 (LOC100260747), VvSUN4 (LOC100254717), VvSUN5 (LOC100854442), VvSUN6 (LOC100254187), VvSUN7 (LOC100243111), VvSUN8 (LOC100240779), VvSUN9 (LOC100244856), VvSUN10 (LOC100266234), VvSUN11 (LOC100247418), VvSUN12 (LOC100256590), VvSUN13 (LOC100253695), VvSUN14 (LOC100265924), VvSUN15 (LOC100266890), VvSUN16 (LOC100241183), VvSUN17 (LOC100241471), VvSUN18 (LOC100256816), VvSUN19 (LOC100245132), VvSUN20 (LOC100245639), VvSUN21 (LOC104882151), VvSUN22 (LOC100255609), VvSUN23 (LOC100245291), VvSUN24 (LOC100263965), VvSUN25 (LOC100246947); *Arabidopsis thaliana*, AtIQD15 (AEE78534.1), AtIQD16 (AEE82912.1), AtIQD17 (AEE81939.1), AtIQD18 (AEE27239.1); *C. sativus*, CsSUN2 (XP_011659956.1); *Citrullus lanatus*, (Csa1G575000), *C. lanatus*, ClSUN8 (Cla011257); and *S. lycopersicum*, SlSUN1-SlSUN33 [21].

### Expression investigations

The Trizol^®^ reagent (Thermo Fisher Scientific, USA) was employed to isolate total RNA in compliance with the guidelines stipulated by the manufacturer. From each sample, 1 μg of total RNA was obtained and subjected to treatment with RNase-free DNase (Vazyme, Nanjing, China) to eliminate any remaining genomic DNA, followed by conversion to cDNA with the aid of the First Strand cDNA Synthesis Kit (Thermo Fisher Scientific, USA). Subsequently, we conducted qRT–PCR utilizing SYBR Premix ExTaq (Takara Biotech, Japan) on a Quant Studio™ 5 System (Thermo Fisher Scientific, USA). Normalization of the targeted gene was carried out by using the *VvActin* and *SlActin* genes. Next, the 2^– ∆∆Ct^ method was employed to compute gene expression levels. As depicted in the corresponding figures, all qPCR tests for each biological replicate were carried out with three technical replicates. Supplementary Data Table S2 displays the primer sequences that were utilized for qRT–PCR.

### Isolation of the *VvSUN* and *VvSUN* promoter

Information on DNA and protein sequences was obtained from the NCBI database. The procedures reported by Zheng *et al*. [42] were utilized for DNA extraction, isolation of total RNA, first-strand cDNA synthesis, and DNase I treatment. The *VvSUN* (LOC100253695) cDNAs were isolated from six different grape cultivars during the pre-bloom phase by RT–PCR based on primers *VvSUN*-F/*VvSUN*-R (Supplementary Data Table S2) before cloning them into the pEASY^®^-Blunt Cloning Vector (TransGen). The *VvSUN* promoter (−1833 bp to ATG) from different cultivars was amplified by primers *VvSUNpro*-F/R from grape DNA, followed by cloning into the pEASY^®^-Blunt Cloning Vector (TransGen). Several clones were randomly chosen and verified by sequencing. The DNAMAN program (Lynnon Biosoft, San Ramon, CA, USA) was employed to assess sequence alignment. Supplementary Data Table S2 gives a complete list of all of the primer pairs that were utilized.

### Generation of *VvSUN* overexpression transgenic lines

Amplification of the VvSUN gene’s coding sequence was accomplished utilizing PCR, followed by cloning of the resulting fragment into the pEASY^®^-Blunt Cloning Vector and subsequently into the pYH4215 vector utilizing a One Step Cloning Kit (Vazyme, Nanjing, China). A *35S::VvSUN* construction was transformed into ‘Micro Tom’ tomato and tobacco via *Agrobacterium*-induced transformation (strain EHA105) in accordance with the procedures reported by De Jong *et al*. [43]. In half-strength MS media that contained hygromycin (30 mg l^−1^), potential transgenic lines were chosen, and their existence was subsequently verified by RT–qPCR, PCR, and GUS staining. Supplementary Data Table S2 shows the primers that were utilized in the PCR and RT–qPCR experiments. The *T*_4_ generation of the transgenic tomato and the *T*_1_ generation of the transgenic tobacco lines were chosen for physiologic experiments and molecular analyses.

### Yeast one-hybrid assay

The −1833 bp fragment (upstream from the start codon) obtained from the *VvSUN* promoter was amplified from grape genomic DNA and subsequently cloned into the pAbAi vector (Clontech) for the Y1H assay. Co-transformation of recombinant plasmid pGADT7-VvARF6 and pAbAi-VvSUN-pro into yeast strain Y1HGold (Clontech) was performed in accordance with the guidelines provided by the manufacturer. Additionally, transfection of the pGADT7 vector into baits was performed, which served as a negative control. SD/−Ura drop-out medium was used to culture the transformants. After a selection of colonies was made and dilution in sterile ddH_2_O attained an OD600 density of 0.5, 3 μl of suspension was spotted on SD/−Ura/−Leu drop-out containing AbA antibiotic at 30°C. Supplementary Data Table S2 provides detailed information on the primers that were utilized in this investigation.

### GUS activity analysis

The promoter of *VvSUN* was ligated into the pBI121-GUS vector before infusion into GV3101 to allow temporary expression in grape leaves. Subsequently, the grape leaves were grown in an incubator for 24 hours in darkness following vacuum infiltration and then treated with increasing dosages of 10, 50, and 100 mg/l of IAA 48 hours later. The positive and negative controls used in the experiment included the CaMV35S-GUS vector and the water treatment, respectively. GUS activity experiments were performed as previously described [44].

### Subcellular localization of *VvSUN*

The *VvSUN* subcellular localization was determined by the PCR amplification of its full-length cDNA utilizing the primers *VvSUN*-GFP-F/R with incorporated NcoI and SpeI restriction regions and subsequent cloning into the pClone007 Blunt Simple vector (TSINGKE, China). Cloning of the full-length *VvSUN* cDNA into the pCAMBIA1302 vector was done after being verified by sequencing to generate the plasmid that would express the VvSUN-GFP fusion protein when driven by the 35S promoter. pCAMBIA1302-GFP was used as the control vector. The plasmids were added to *Agrobacterium tumefaciens* strain GV3101 following the protocol of Zheng *et al*. [42]. A Zeiss confocal scanning microscope (LSM700) was utilized to monitor the expression of the VvSUN-GFP fusion protein and chlorophyll signals in the infiltrated *N. benthamiana* leaves 3 days after inoculation.

### Quantitative analysis of endogenous IAA and IAA-related compounds

IAA, IAA-Asp, and IPYA used as the standards were procured from Sigma–Aldrich (USA). Endogenous IAA and IAA-related compounds were extracted from ovaries and young fruits of tomatoes by using liquid–liquid extraction and determined by high-performance liquid chromatography-electrospray ionization tandem mass spectrometry (HPLC-ESI-MS/MS) following a published protocol [45]. Data were analyzed using MassHunter Workstation software (Agilent, CA, USA) and the final result was expressed in nanograms per gram FW.

### RNA-seq data analysis

We extracted total RNA from three biologically separate pools of the wild tomato (control) and *35S::VvSUN* line 5 ovaries at 1 WBA, anthesis, and 5 DPA as described above in Expression investigations. The Stranded mRNA-seq kit (Vazyme, Nanjing, China) was utilized to create RNA-seq libraries. Next, the Illumina Novaseq platform (HiSeqTM2500/4000) was utilized for sequence analyses in Vazyme (China). A Trimmomatic (v0.33) was employed to screen the raw reads by eliminating the low-quality and adapter sequences. Mapping of the clean reads to the tomato genome was conducted by using STAR (v2.5.2b) [46]. DESeq (*P*_adj_ <.05, v1.10.1) was employed to analyze the transcript assembly and expression levels of genes [47]. The DEGs affected by the genotype and developmental phase were clustered utilizing *K*-means in R [48]. Co-expression networks were created by using the WGCNA (v1.29) package in R and Cytoscape software (v3.9.1) [49]. The expression data used for WGCNA analysis comprised a total of 22 510 identified genes in the present study from the 18 datasets, and the trait data included longitudinal diameter, transverse diameter, FSI, IAA, IAA-ASP, IPYA, and fruit development stage. Clustering of the genes identified from *K*-means and WGCNA were performed using GO (GOSeq, v1.22) [50] and KEGG (KOBAS, v2.0) [51] enrichment analyses. The annotation file was downloaded from the tomato database (ftp://ftp.ensemblgenomes.org:21/pub/plants/release-47/fasta/solanum_lycopersicum/).

Based on the gene’s annotations, transmembrane transport pathway genes and auxin-related genes implicated in its metabolic activities, signal transduction, and polar transport were identified from DEGs. Excel was utilized to perform pairwise comparisons of the expression values between the *35S::VvSUN* and control at each developmental stage and the findings were subjected to log_2_ transformation.

## Acknowledgements

This work was supported by the National Natural Science Foundation of China (Grant No. 31972384 and 31901975), China Agriculture Research System of MOF and MARA, the National Key Research and Development Program of China (2020YFD1000204), the China Postdoctoral Science Foundation (2019 M651858), the Jiangsu Agricultural Industry Technology System (JATS [2021]450), and the Jiangsu Key Agricultural Project for New Cultivars Innovation (PZCZ201723). The authors thank Proof-Reading-Service (https://www.proof-reading-service.com/) for language editing, which has improved the manuscript.

## Author contributions

H.Z. and J.M.T designed the study. H.Z., Y.D., and H.L.N conducted the related experiments and data analysis and wrote the manuscript. J.L., X.Y., Y.G.Z., B.H., W.W., and L.Y.H. participated in the experiments. L.N.Y. and J.M.T. reviewed and revised the manuscript.

## Data availability

The RNA sequencing datasets generated in this study have been deposited in the National Genomics Data Center with the accession number PRJCA009128 (https://ngdc.cncb.ac.cn/). Other data supporting our findings are available in the manuscript file or from the corresponding author upon request.

## Conflict of interest

The authors declare no competing interests.

## Supplementary data

Supplementary data is available at *Horticulture Research* online.

## Supplementary Material

supp_data_uhac200
