# Supplementary material for: VvSUN may act in the auxin pathway to regulate fruit shape in grape
Source: Hortic Res. 2022 Sep 6;9:uhac200. doi: 10.1093/hr/uhac200 (PMC9647697; doi:10.1093/hr/uhac200)
Supplement: supp_data_uhac200 [file supp_data_uhac200.zip › supplementary_files_8.20_clean version.pdf]

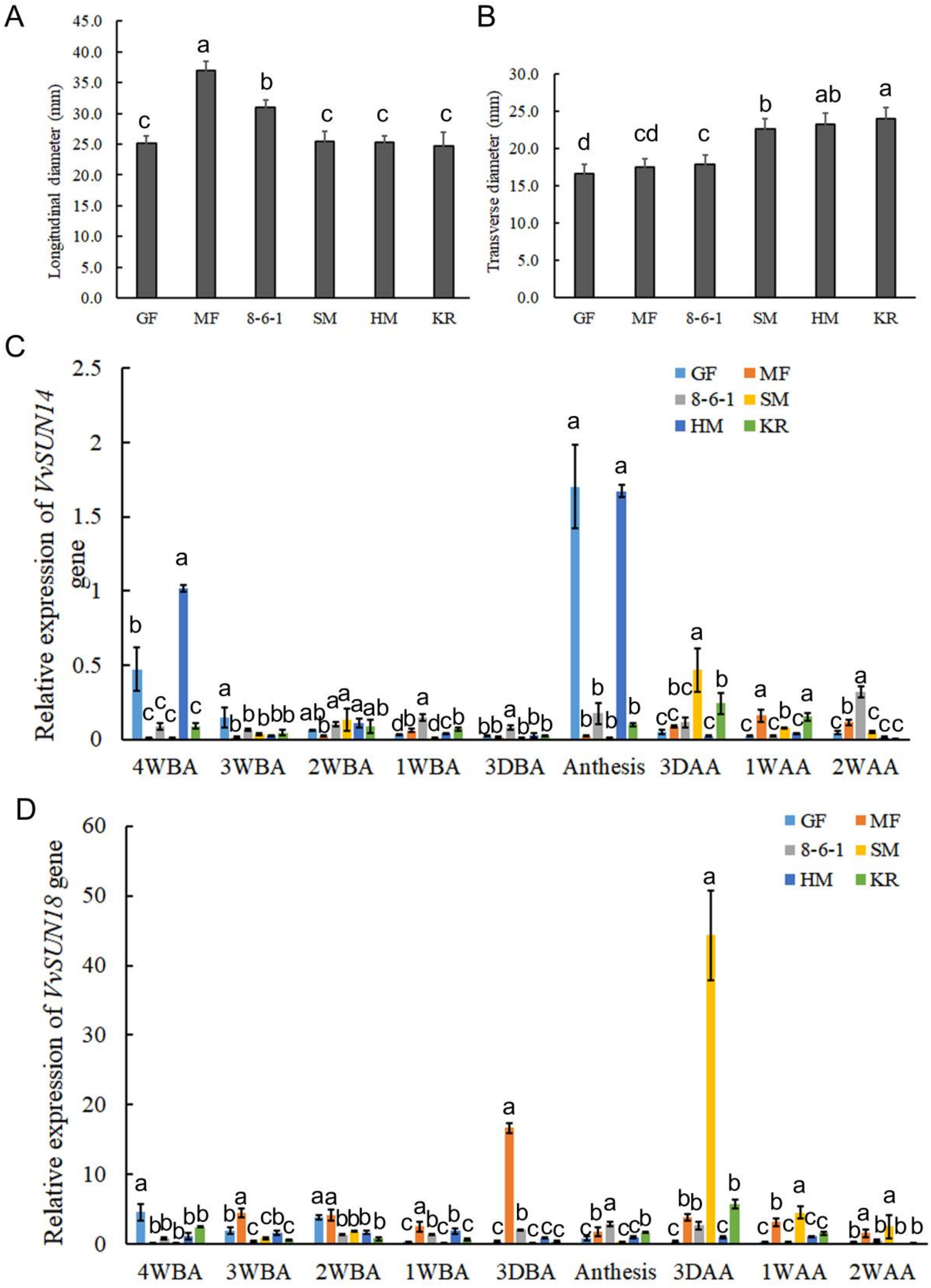

Fig. S1. Morphological assay of different grape varieties and expression analysis of *VvSUN14* and *VvSUN18* genes. (A) Longitudinal diameter and (B) transverse diameter analysis of mature berries of different grape varieties. Lower case letters ($P \leq 0.01$) represent a significant difference among different types, as determined by the Student's *t*-test. (C) The transcription of *VvSUN14* and (D) *VvSUN18* at various phases of ovary/fruit development was identified utilizing qRT-PCR. Normalization of the expression levels was

conducted using the *VvActin* transcript, and the *VvSUN14* and *VvSUN18* expression in HM at the 4WBA stage was set as the control group to compute relative expression levels, respectively. Experimental studies were replicated biologically 3 times, and the data were presented as the mean ± standard deviation (SD). Lower case letters ($P \leq 0.01$) represent a significant difference among different stages, as determined by the Student's *t*-test.

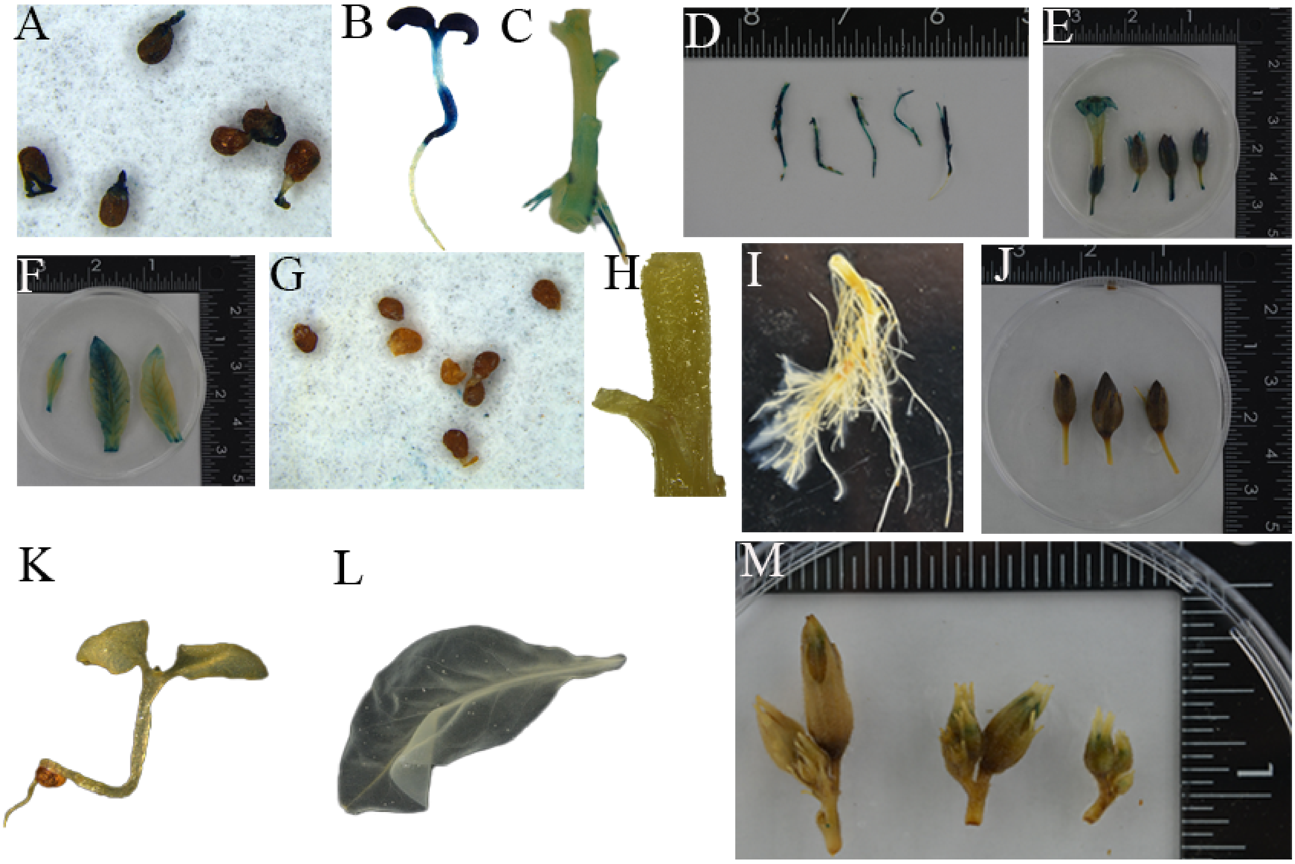

Fig. S2. Histochemical GUS staining of *pVvSUN::GUS* tobacco lines. (A-F) *p35S::GUS* tobacco lines as the control; (G-M) *pVvSUN::GUS* tobacco lines. (A) and (G), germinated seeds; (B) and (K), a 15-day-old seedling; (C) and (H), stem; (D) and (I), root, (E) and (J), opening flowers and seeds; (F) and (L), leaves; (M), different stages before anthesis.

MGKKRSWFSLVKRIFISEAKEKEEKKPKSWRCLFTRFKLKQCPTLAAPRRTLTEARAEQRKHALTVALATAAAAEAAVAAARAA

AEVVRLTCSPPSYYHTCDKRNQNLAAIKIQTAFRGYLARKALQALKGLVRLQALVRGQIVRRQAITKLKCLPSTANTRAQVNIGG

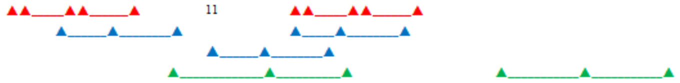

VLTTEETYKDGNNRKFLRPKKECGGREIKAYVIEQLEGSGQRSWDYNILSQEDVETIWLRKQEALIRRERMKKYSSSHRERINAQ

MPEETEPYKENGRQSCQLVRWMDSMEHKRKEAENSKAAADSNLLNGHINGTTNIELRNGWKQNSTEGSDMPFSLPRRSFCHRK

HNSVIDDSSFPSSPVFPTYMAATESAKAKARALSTPKQRLGFLDSCFDQSSPYTNRPSFWSSLNGESISSGRSGYSWQNTVSMKS

LN

Fig. S3. The IQ67 domain of the *VvSUN* gene was subjected to a motif analysis. IQ motifs (red); the 1-8-14 motifs (green); the 1-5-10 motifs (blue).

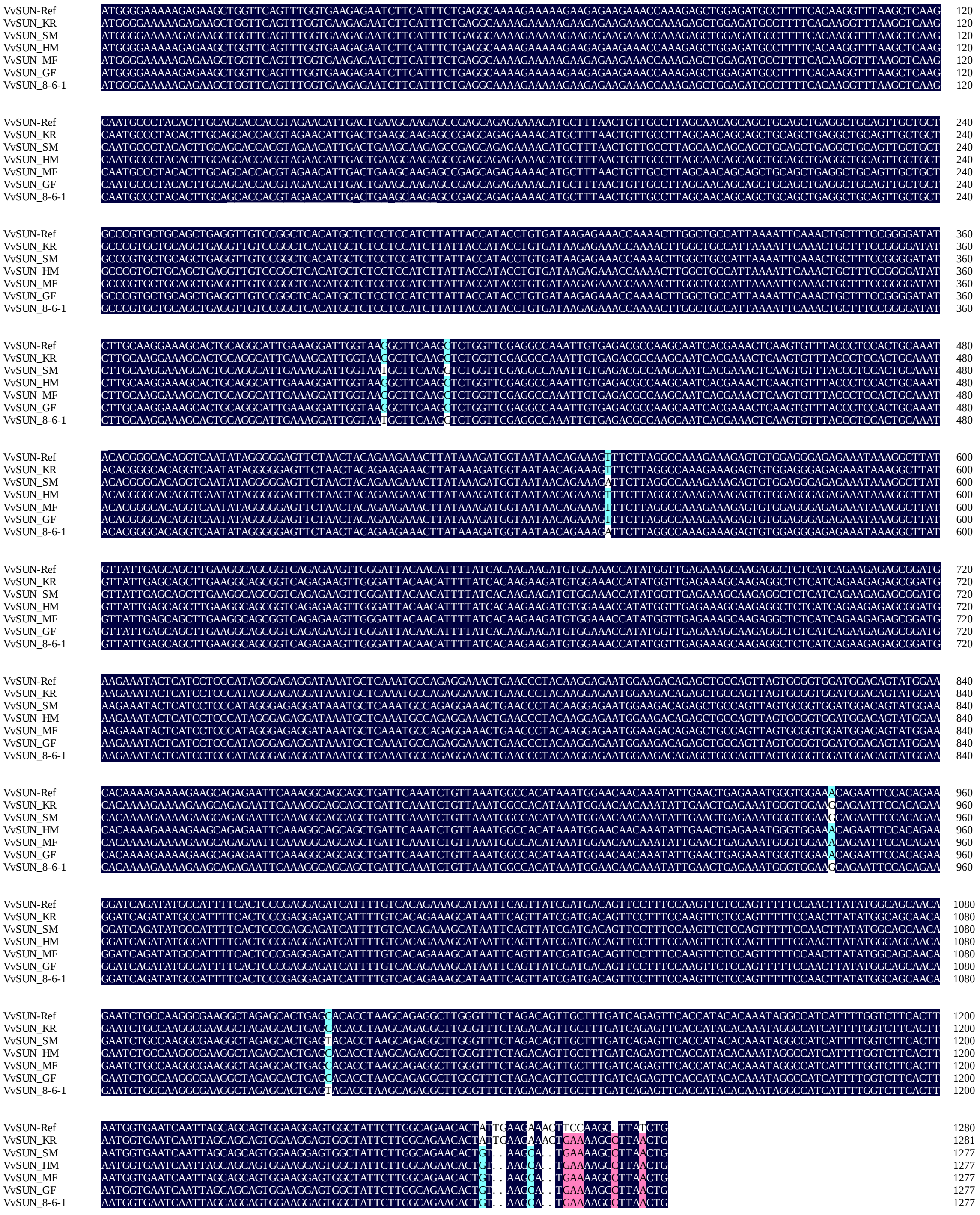

Fig. S4. Multiple sequence alignments of *VvSUN* gene among different grape cultivars. KR: ‘Kourgan Rose’, SM: ‘Shine-Muscat’, HM: ‘Houman’, MF: ‘Minicure Finger’, GF: ‘GoldFinger’ and 8-6-1 (‘Beni Pizzutello’ seedling).

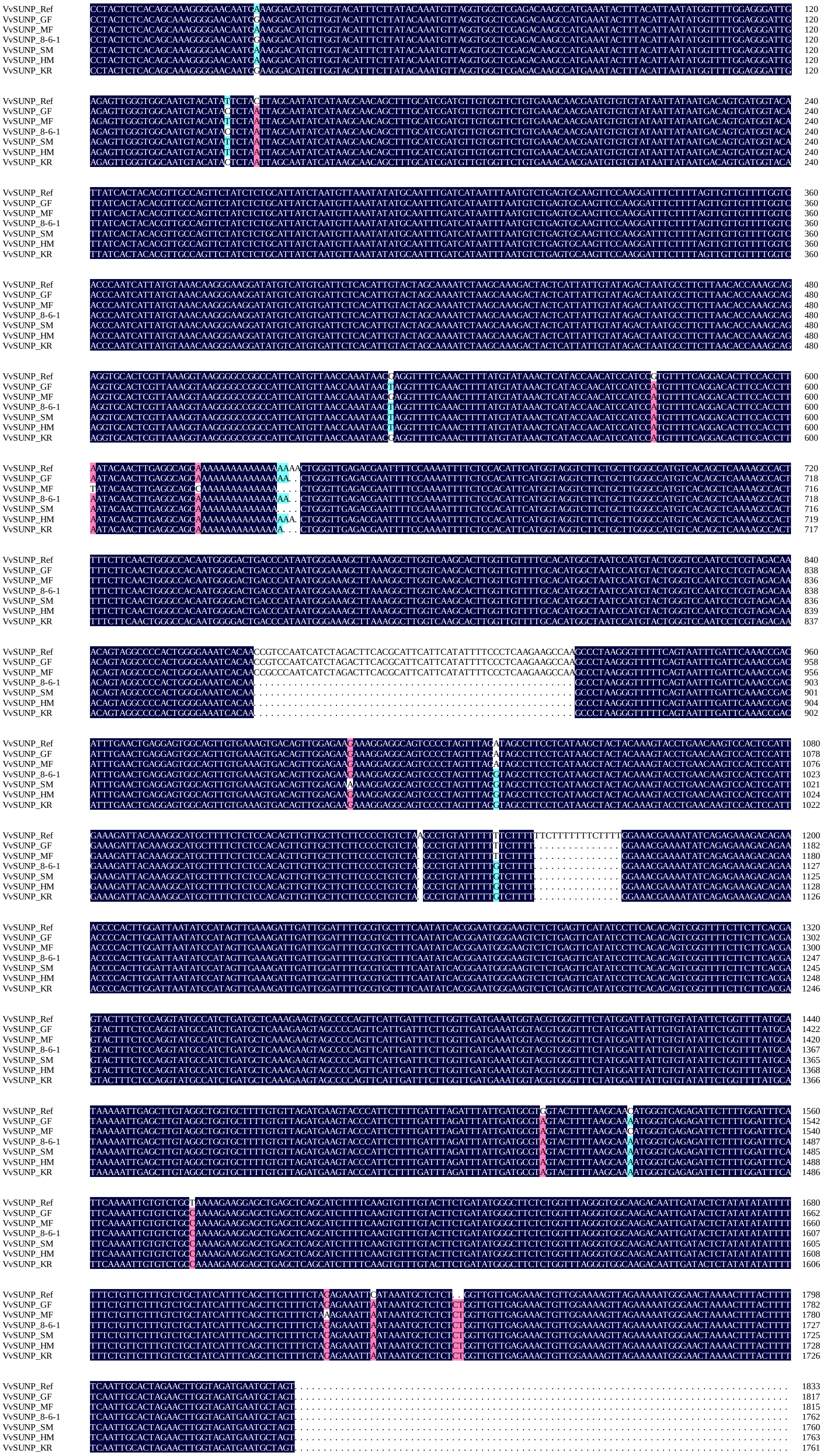
VvSUNP_Ref
VvSUNP_GF
VvSUNP_MF
VvSUNP_8-6-1
VvSUNP_SM
VvSUNP_HM
VvSUNP_KR

Fig. S5. Multiple sequence alignments of *VvSUN* gene's promoter (from -1833 bp to ATG) among different grape cultivars. KR: 'Kourgan Rose', SM: 'Shine-Muscat', HM: 'Houman', MF: 'Minicure Finger', GF: 'GoldFinger' and 8-6-1 ('Beni Pizzutello' seedling).

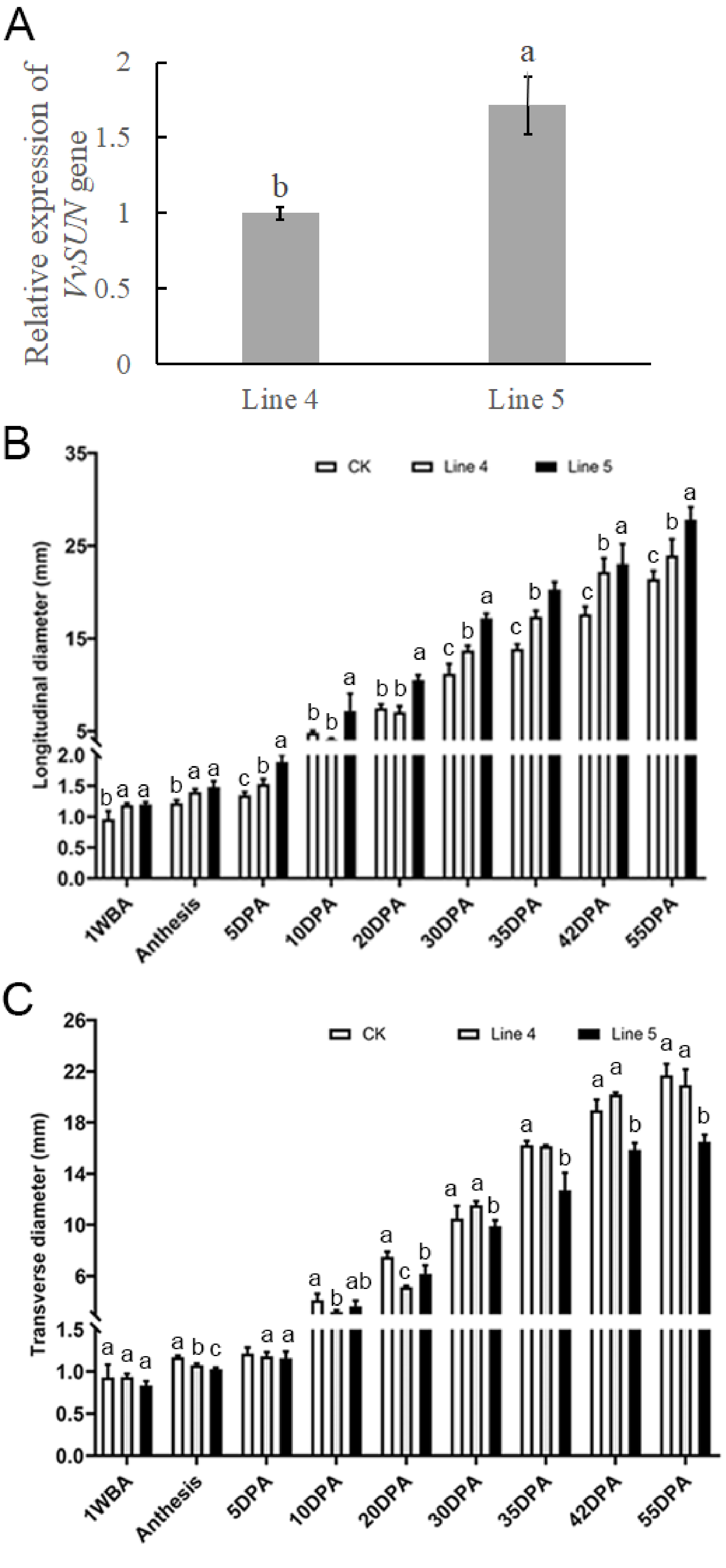

Fig. S6. Phenotypic characteristics of *35S::VvSUN* tomatoes. (A) The expression level of *VvSUN* gene in transgenic tomato lines. Normalization of the expression levels was conducted using the *SlActin* transcript, and line 4 was set as the control group to compute relative expression levels. Experimental studies were replicated biologically 3 times, and the data are presented as the mean ± SD. Evaluation and statistical analyses of ovary/fruit length (B) and width (C) (mm) of CK and *35S::VvSUN* tomatoes at the fruit development stages. Data are shown as the mean ± SD. Lower case letters ($P \leq 0.01$) represent a significant difference among different genotypes, as established by the Student's *t*-test.

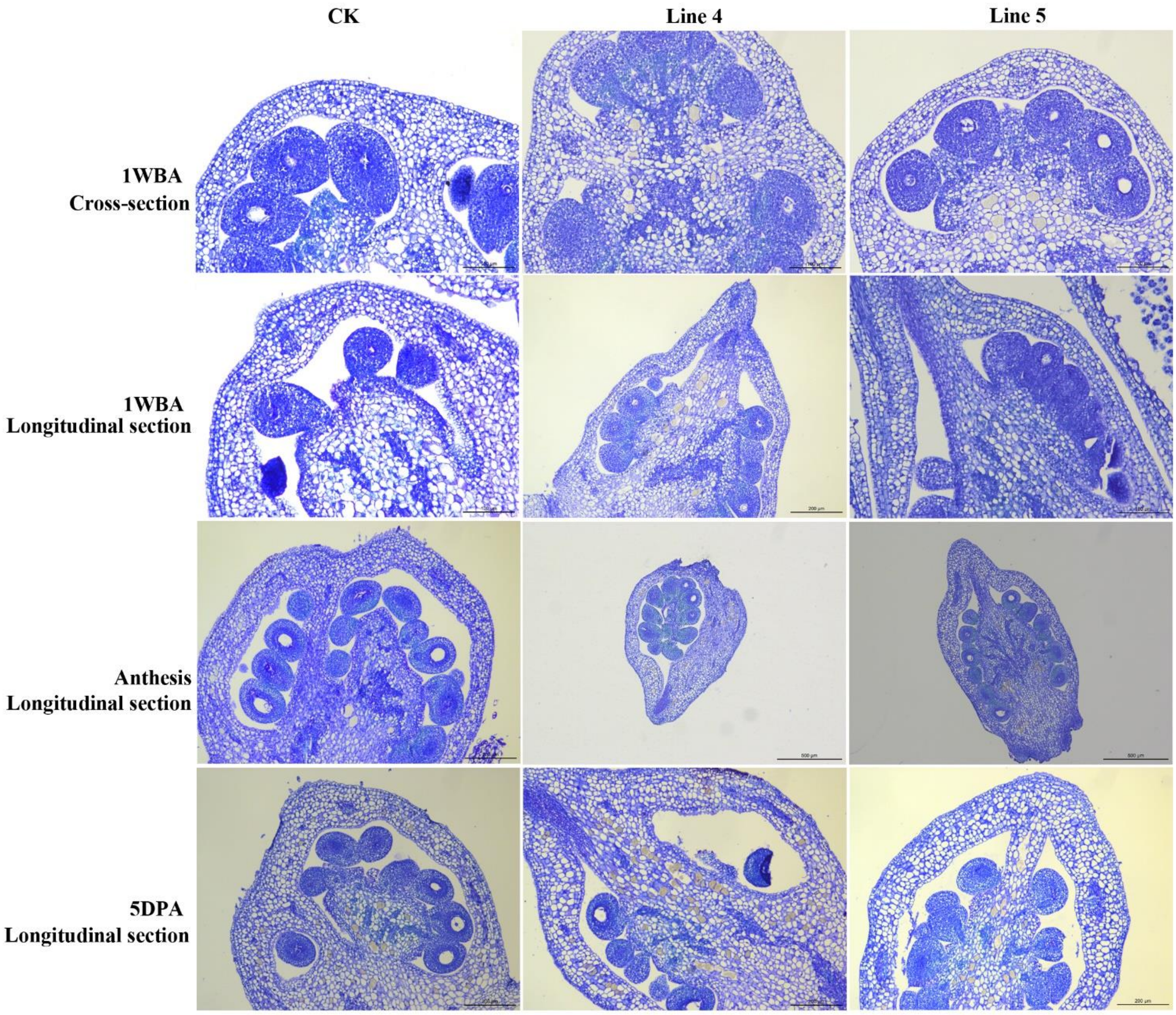

Fig. S7. Bright-field images of ovaries captured at distinct stages of development from *35S::VvSUN* transgenic tomato and control plants.

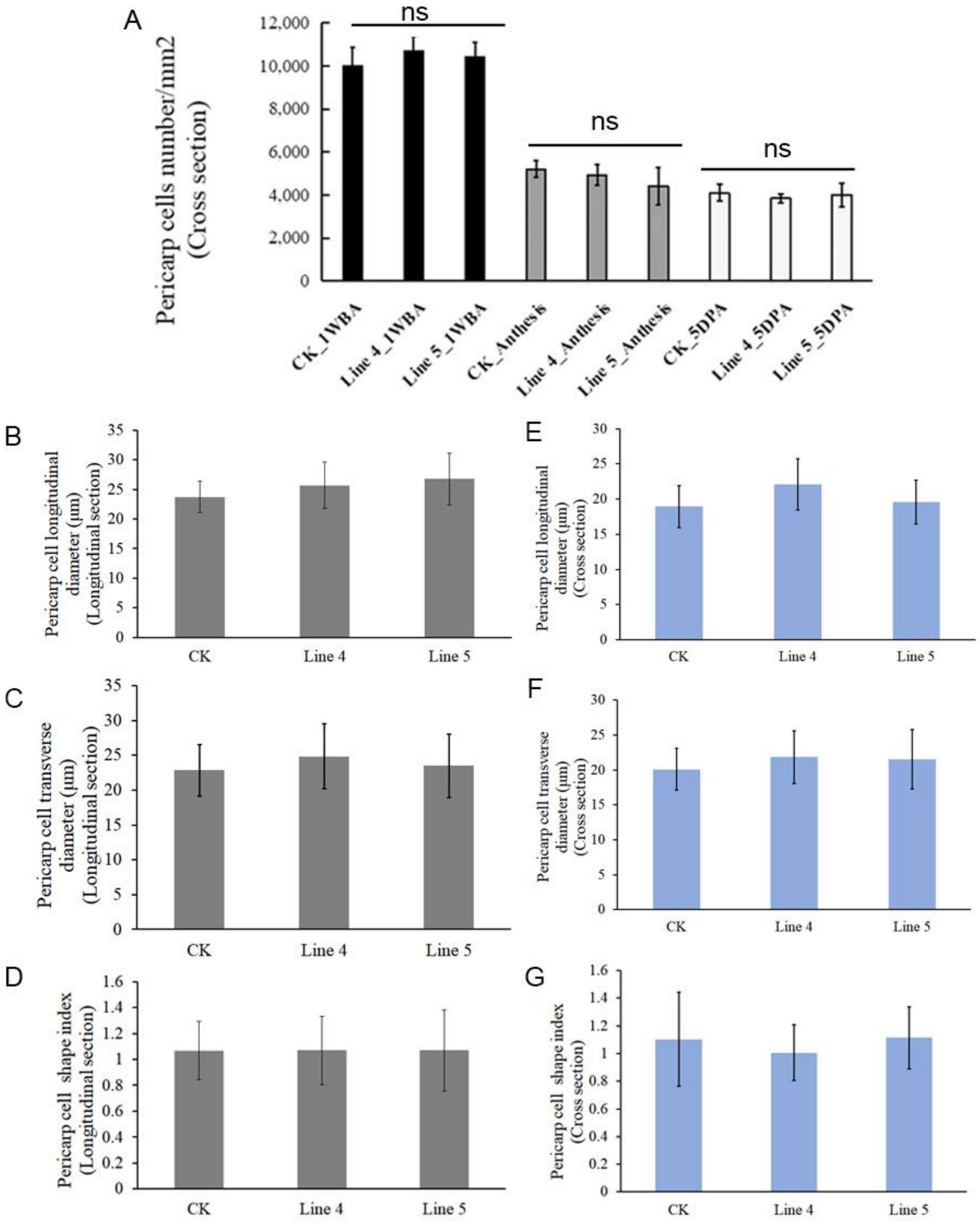

Fig. S8. Comparison of pericarp cell numbers (cross section) in three stages (A) and cell shapes in CK and lines 4 and 5 at 1WBA stage. Data are shown as the mean ± SD. Significant difference among different genotypes were established by the Student's *t*-test.

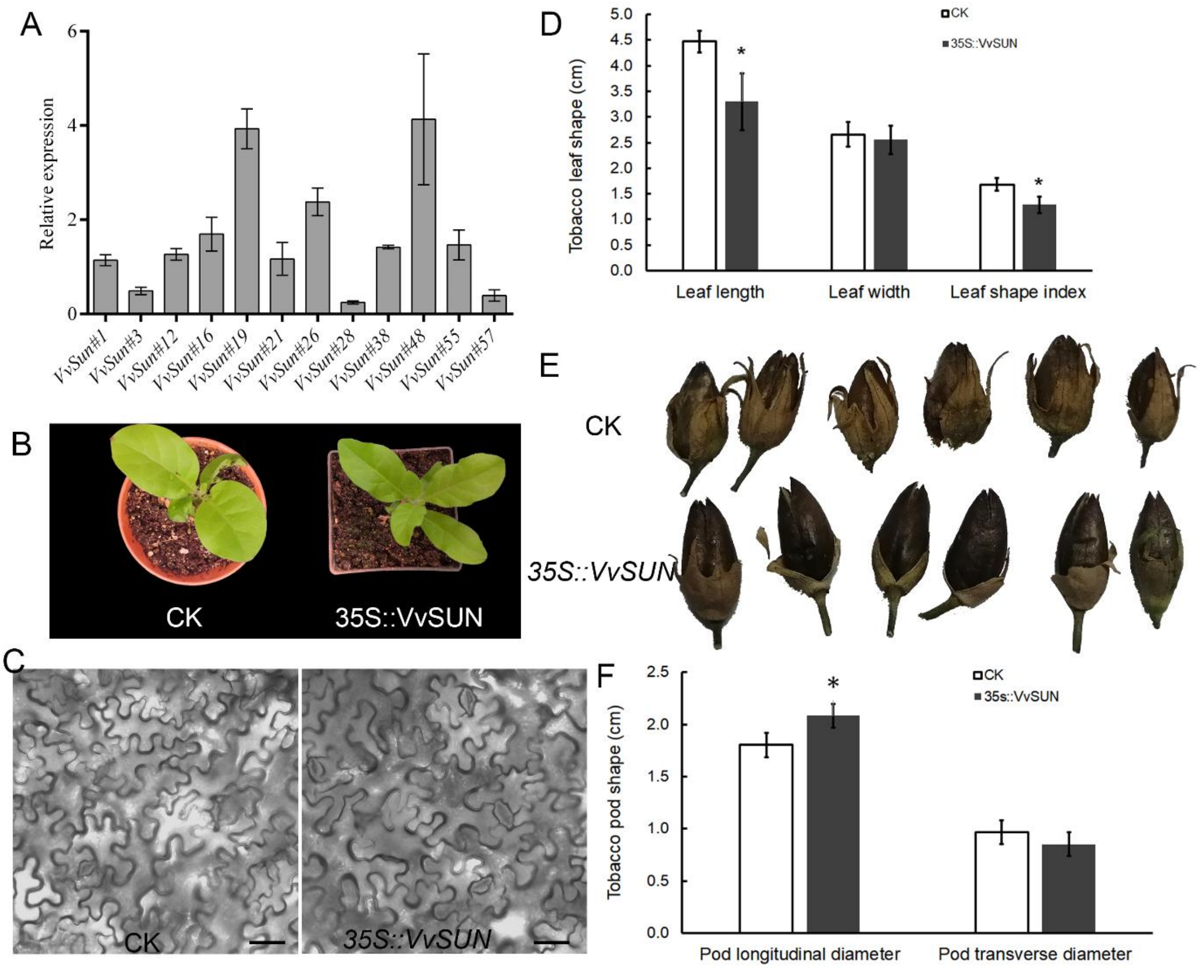

Fig. S9. Phenotypic characteristics of the *35S::VvSUN* transgenic tobacco. (A) *VvSUN* expression in the different transgenic lines was analyzed by qRT-PCR. (B) Leaf morphology of CK and *35S::VvSUN* transgenic tobacco. (C) Single optical sections are shown for epidermal cells (adaxial side) of the fourth leaf in the seedlings. Bar = 50 mm. (D) Assessment and statistical analyses of leaf width and length (cm), leaf shape index (length/width ratio) of CK and *35S::VvSUN* transgenic tobacco. (E) Pod morphology of CK and 35S::VvSUN transgenic tobacco. (F) Assessment and statistical analyses of the pod width and length of CK and *35S::VvSUN* transgenic tobacco. Outcomes are presented as the mean ± SD, and statistical analysis was done by computing the P-value utilizing the Student's *t*-test; *, P <0.05.

A

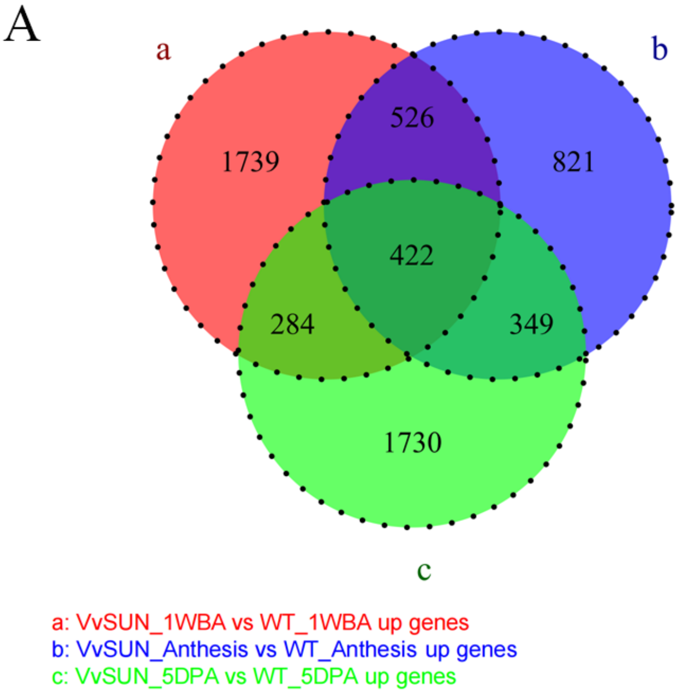

B

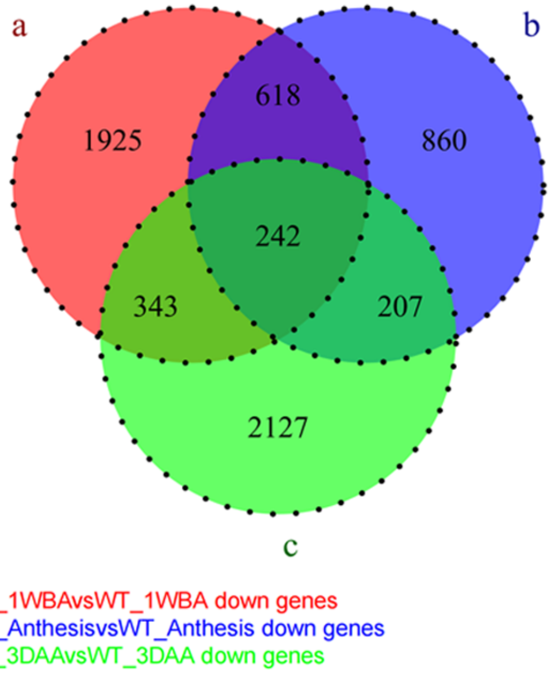

Fig. S10. Venn diagrams showing the numbers of (A) up- and (B) down-regulated DEGs among three stages in *35S::VvSUN* transgenic tomato.

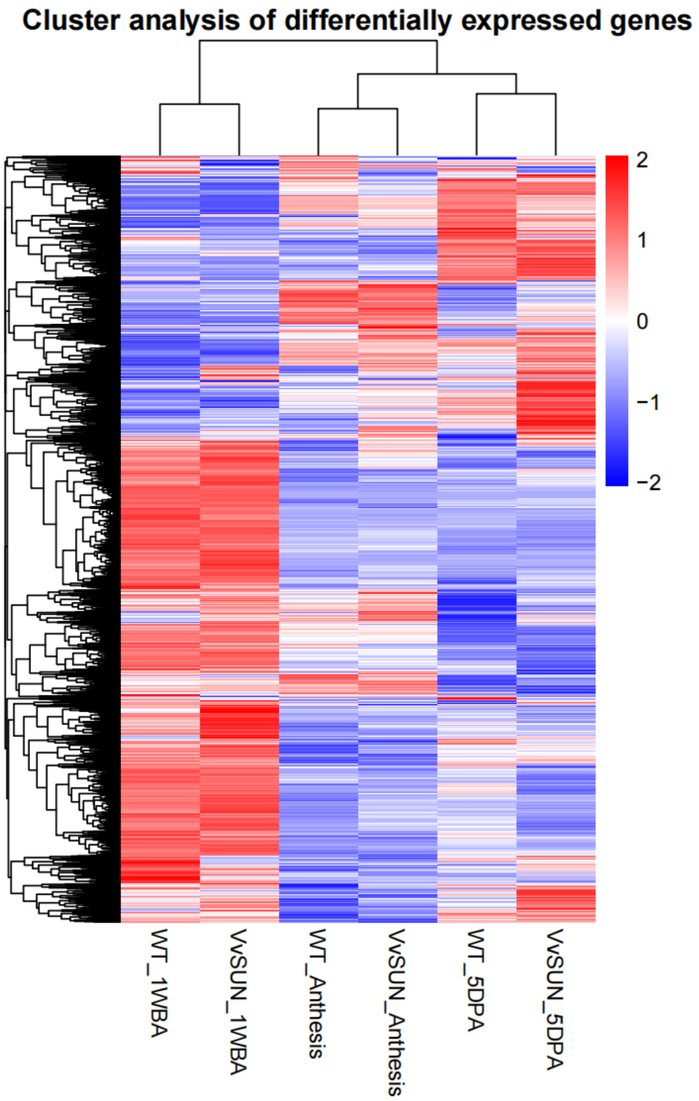

Fig. S11. Heatmap of the DEGs according to the adjusted *P*-value and $\log_2$(ratio). Gene expression is shown by the colors red and blue. Red signifies elevated gene expression levels, while blue shows decreased gene expression levels.

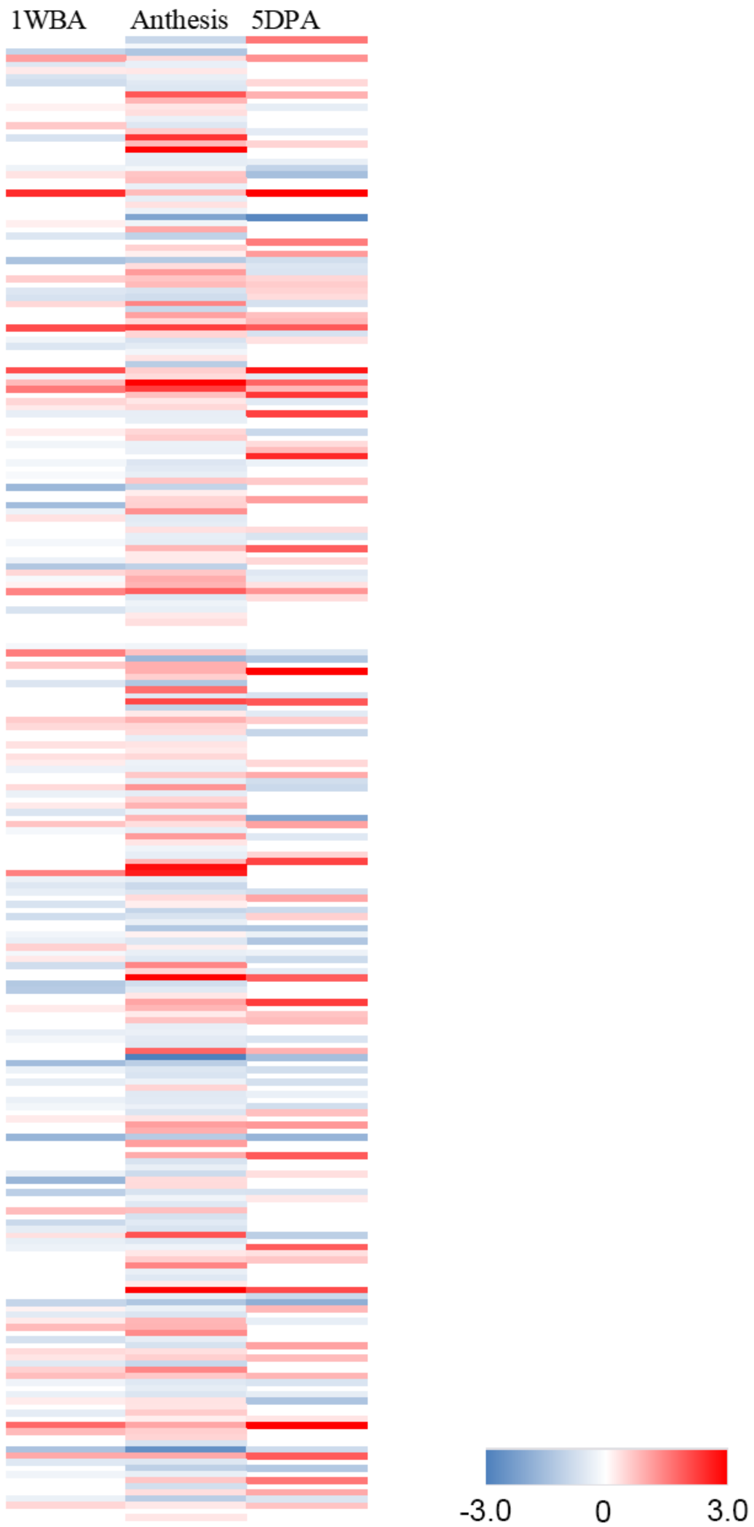

Fig. S12. The expression changes of different transmembrane transport pathway-related genes. The color legend represents the log_2-fold change in the

VvSUN/CK ratio. Positive values were higher in *35S::VvSUN* transgenic tomato. Negative values were higher in the control plant.

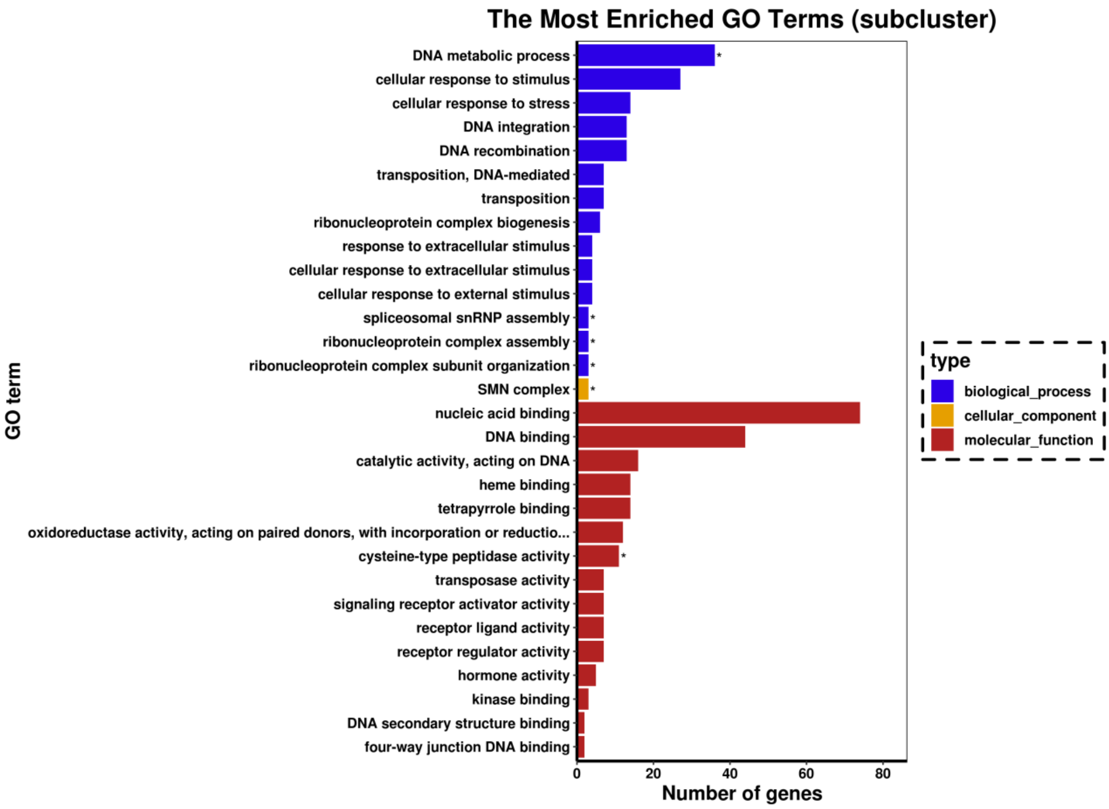

Fig. S13. GO enrichment analysis of the DEGs in *35S::VvSUN* transgenic tomato, showing contrasting expression trends in comparison with the control tomato.

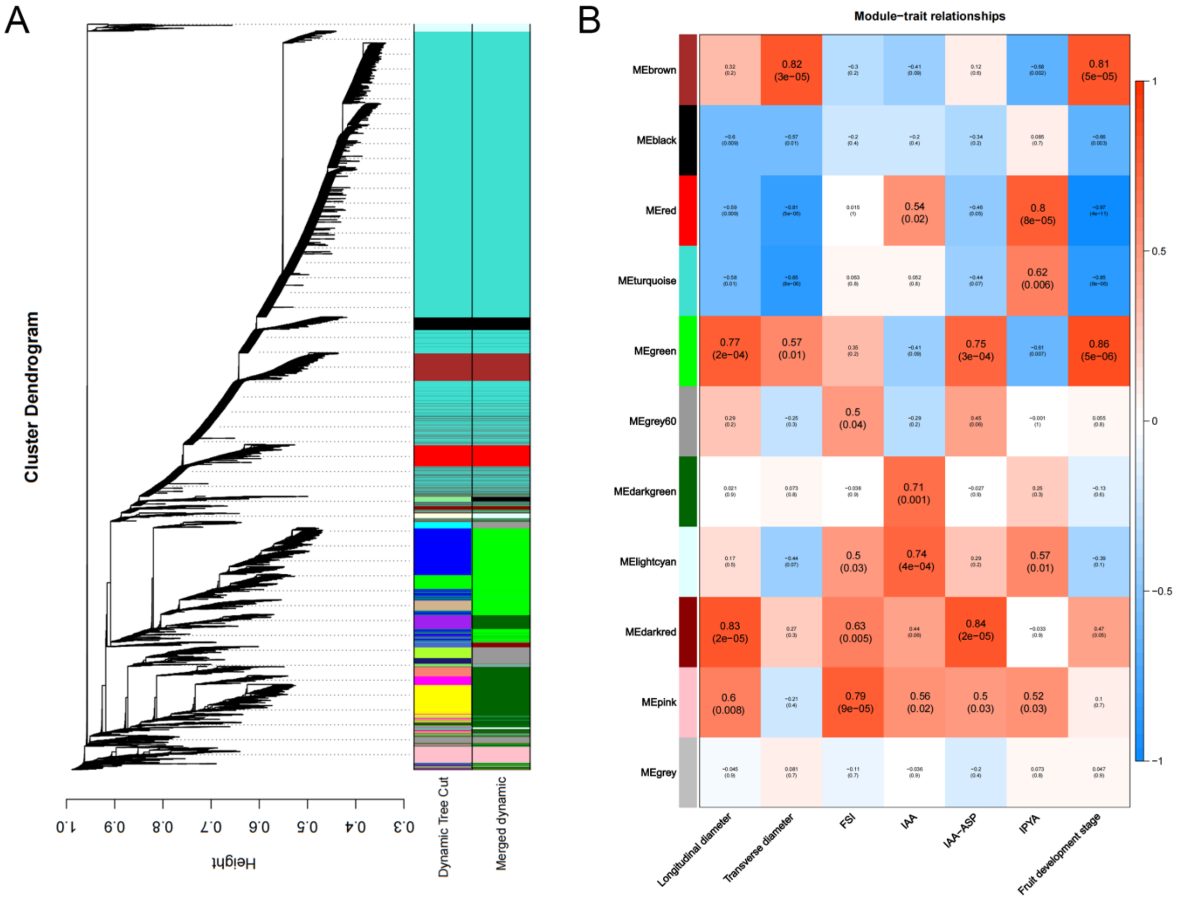

Fig. S14. Network analysis dendrogram showing modules identified by WGCNA. A. Dendrogram plot with color annotation. B. Module-traits correlations and corresponding P-values. The left panel shows the 11 modules. The color scale on the right shows module trait correlation from −1 (blue) to 1 (red).

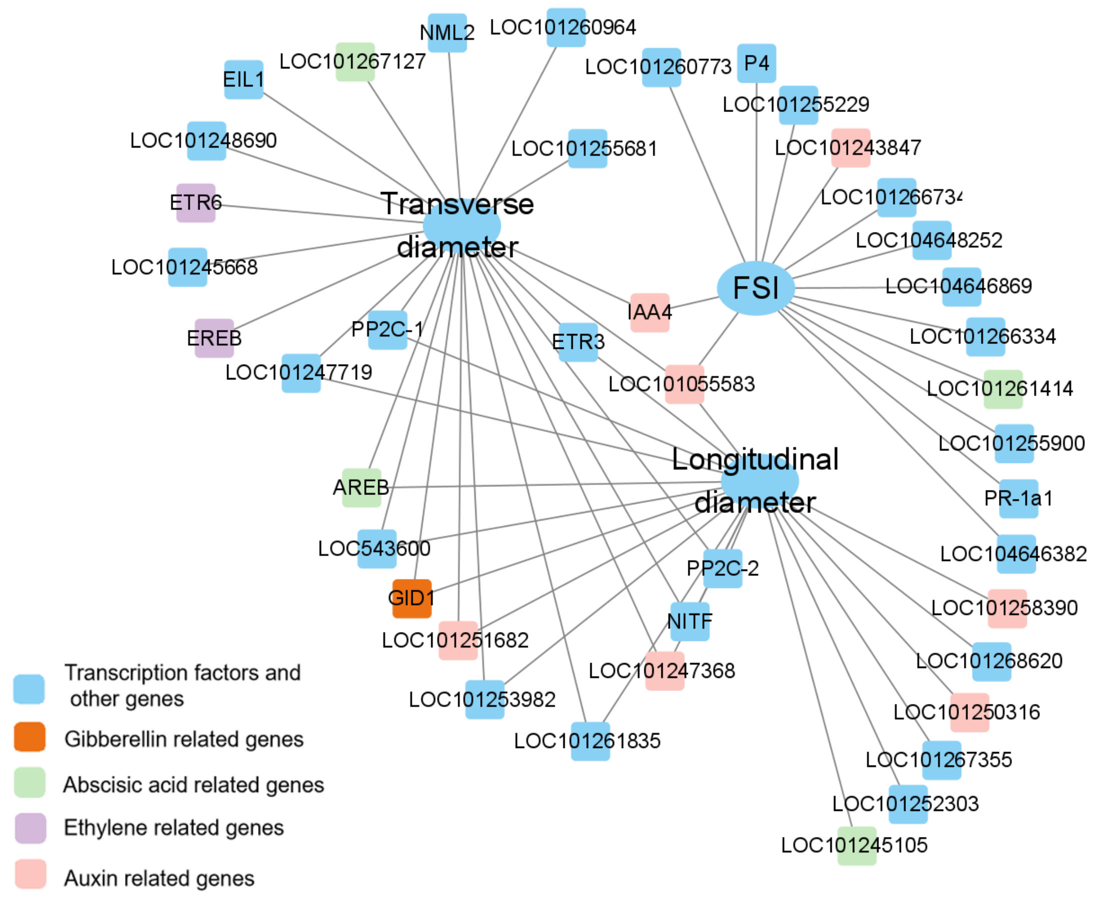

Fig. S15. Computed regulatory network of the 41 genes in plant hormone signal transduction pathways, The genes were clustered based on their trait modules and were presented in different colors according to their gene function annotation. (see Supplementary Table S7)

Table S1 List of plant cis-acting regulatory elements in *VvSUN* promoter sequence

Classification	Name	Number	Function
Specific regulatory element	MYB1AT	6	MYB binding site
	MBS	4	MYB binding site involved in drought-inducibility
	CAT-box	2	Cis-acting regulatory element related to meristem expression
	O2-site	2	Cis-acting regulatory element involved in zein metabolism regulation
	TCA-element	1	Cis-acting element involved in salicylic acid responsiveness
Plant hormone responsive element	ARFAT	1	Auxin-responsive element
	ABRE	3	Abscisic acid-responsive element
	ABRE3a	1	Abscisic acid-responsive element
	ABRE4	1	Abscisic acid-responsive element
	P-box	1	gibberellin-responsive element
Light responsive element	Box 4	3	Light responsive element
	G-Box	2	Light responsive element
	GT1-motif	1	Part of a light responsive element
	I-box	1	Light responsive element
Core transcriptional element	CAAT-box	31	Common element in promoter
	TATA-box	21	Core regulation site of transcription initiation

Table S2 The primer sequences that were employed in this research

Genes	Primer name	Sequence (5’-3’)	Usage	GenBank accession No.
VvSUN	F	ATGGGGAAAAAGAGAAGCTGGTTCA	cDNA cloning and genomic DNA cloning of *VvSUN* gene	LOC100253695
	R	TCAGTTAAGGCTTTTCATGCTTACAGTG		
VvSUN_CDS	F	ATGGGGAAAAAGAGAAGCTGGTTCA	Coding sequence cloning of *VvSUN* gene	
	R	TCAGTTAAGGCTTTTCATGCTTACAGTG		
MQSUN	F	CGGGATCCATGGGGAAAAAGAGAAGCTGGTTCA	Construction of plant binary expression vector for transformation	
	R	CGAGCTCTCAGTTAAGGCTTTTCATGCTTACAGTG		
VvSUN-PRO	F	CAGCAACCCTTTTCTCTCAAGTC	Cloning of *VvSUN* gene promoter	
	R	CCAAAATTAAAGAATTTCGCCTGCT		
VvSUN-GFP	F	CCCATGGATGGGGAAAAAGAGAAGCTGG	Subcellular localization of *VvSUN* gene	
	R	CTAGACTAGTGTTAAGGCTTTTCATGCTTACA		

VvARF6	F	GAATTCATGAGGTTGTCTCCTGCTGG	Coding sequence cloning of *VvARF6* gene	LOC100242923
	R	GGATCCTCAACCTCTATGAAGGCTTGAGC		
proSUN-AbAi	F	CCCGGGAGCCAAAATTAAAGAATTTCGCC	Yeast one hybrids	
	R	AAGCTTGGATAATGATGGAAGATGGCCA		
VvActin	F	ATGGCAGACGGAGAGGATATTCA	Housekeeping gene	
	R	GCCTTTGCAATCCACATCTGCTG		
VvSUN-YG	F	CAGCAACAGAATCTGCCAAG	Expression of *VvSUN* gene	
	R	CCTTCCACTGCTGCTAATTG		
VvSUN14-YG	*F*	GATCTACCCCGCGCTTGAA	Expression of *VvSUN14* gene	*LOC100265924*
	R	CTGACTGGGCCTTAACAGCA		
VvSUN18-YG	*F*	AAGGTCAAGACGAGTGGCAG	Expression of *VvSUN18* gene	*LOC100256816*
	R	ATGCATGTCCAGGCTCCAAA		
SlActin	F	GAAATAGCATAAGATGGCAGACG	Housekeeping gene	NM_001330119
	R	ATACCCACCATCACACCAGTAT		
